# Early and Real-Time Detection of Seasonal Influenza Onset

**DOI:** 10.1371/journal.pcbi.1005330

**Published:** 2017-02-03

**Authors:** Miguel Won, Manuel Marques-Pita, Carlota Louro, Joana Gonçalves-Sá

**Affiliations:** 1 Instituto Gulbenkian de Ciência, Oeiras, Portugal; 2 Nova Medical School, Universidade Nova de Lisboa and Saude 24, Lisbon, Portugal; The Pennsylvania State University, UNITED STATES

## Abstract

Every year, influenza epidemics affect millions of people and place a strong burden on health care services. A timely knowledge of the onset of the epidemic could allow these services to prepare for the peak. We present a method that can reliably identify and signal the influenza outbreak. By combining official Influenza-Like Illness (ILI) incidence rates, searches for ILI-related terms on Google, and an on-call triage phone service, Saúde 24, we were able to identify the beginning of the flu season in 8 European countries, anticipating current official alerts by several weeks. This work shows that it is possible to detect and consistently anticipate the onset of the flu season, in real-time, regardless of the amplitude of the epidemic, with obvious advantages for health care authorities. We also show that the method is not limited to one country, specific region or language, and that it provides a simple and reliable signal that can be used in early detection of other seasonal diseases.

## Author Summary

Influenza, generally referred to as the flu, is a common infectious disease that affects millions of people. Every year, we expect this seasonal disease to occur during the Winter, but exactly when it will start and how severe it will be is not known. This places a strong burden on health services, as often the spread can be felt as very fast and emergency rooms become flooded with patients. With this work, we propose a new method that identifies the beginning of the yearly flu season. This is done by using several different data sources, including searches for flu-related symptoms on Google and phone call logs to a specialized medical phone service. These data sources, together with our method, can provide a daily or weekly report, making it much faster than current methods, which require lab testing or centralized medical reports. Our method was applied to different European countries and can anticipate current official alerts by several weeks.

## Introduction

Seasonal influenza is a worldwide infectious disease estimated to be the cause of 3 to 5 million cases of severe illness and up to half a million deaths every year [1], also placing a strong economic burden on health services [2] and [3]. To deal with these epidemics, the beginning of the flu season has to be declared. Following the official alerts, hospital emergency rooms and health care centres activate appropriate flu response protocols and prepare for possible overcrowding. However, and despite occurring yearly, the onset of the influenza outbreaks is unpredictable and this uncertainty poses logistic problems to most public health services, often already under high demand due to excess winter mortality. Therefore, reliable and timely information tools on current influenza activity are of the utmost interests to health services and to health-related decision makers.

In Europe, the European Influenza Surveillance Network (EISN), implemented and coordinated by the European Centre for Disease Control (ECDC), is the leading responsible entity for gathering and reporting data on influenza activity, during each season. This surveillance mechanism relies on a network of sentinel medical doctors, spread throughout all European Union (EU) and European Economic Area (EEA) Member States. These sentinel doctors report on the number of patients with influenza-like illness (ILI) who self-referred to primary health care services, from October to May of each year, and also send samples for laboratory testing. With this information, the ECDC generates a weekly report, referring to the previous week, which includes the estimated number of ILI cases per 100,000 inhabitants, and other indicators such as trend, types and subtypes of circulating influenza viruses, or geographical spread [4], [5]. The EISN-ILI method is arguably one of the best surveillance systems in the world and a systematic source of reliable data. However, it faces several challenges. First, only an unknown sized sample of those with ILI seek medical care, and this sample can change depending on the circulating virus subtype, from season to season and from country to country; second, the number of medical professionals participating in the sentinel network is small (1–5% of physicians working in the country or region [5]), which can result in low statistical significance and unpredictability; third, even if consultations happened with no delay, the data would be available at best with one week lag. Thus, this system can lead to under-reporting, especially early in the season, when both medical doctors and the general population haven’t been alerted to the presence of a circulating Influenza-Like Virus. This means that between the actual onset of the seasonal epidemics and the official alert, several weeks can elapse.

These limitations have been recognized by others and past studies have focused on forecasting the ILI incidence rate independently of clinical consultations, by using data from on-line volunteer participants [6], ILI-related queries on Google [7], Wikipedia logs [8] or a combination of several data sources [9]. All these systems were designed to give the best, real-time, ILI rate estimates.

However, and irrespective of the data source used, these studies often focus on the USA or in one single country, and might be difficult to generalize to different regions. Moreover, influenza dynamics research [10] is often mainly concerned with simulating the flu season’s number of cases, as well as the peak’s timing, neglecting timely onset identification.

We argue that, from the health policy stand point, it is fundamental to be able not only to track changes in incidence rate, but also to accurately know when the flu season has started, as a major concern with the outbreak of influenza is the immediate over-burden of health resources. An early detection of the flu onset could a) anticipate the provision or reinforcement of health professionals and facilities; b) confidently advise the generally healthy population to stay home, redirecting them from the likely to-be crowded emergency services [11]; and c) signal the entire EINS network, possibly even improving the surveillance system.

In fact, the cited 2014 review [10] listed 24 papers that focus of seasonal influenza forecasting or that could be applied to seasonal data. Several identify onset prediction as an important goal [12][13] but only one [14] tries to do on onset prediction, although not in real time. This has been the case of few other studies, that develop methods to identify the onset, or show that different systems and data sources could be used to it, either with real data or just by testing different models, such as [15][16][17][18][19], and particularly [20], which focuses on the potential of GFT. But, to our knowledge, no other work has focused on developing and testing a real-time onset detection system.

Thus, in this study we present a different approach and describe a method to identify the onset of the seasonal influenza epidemics, using alternative and real-time data-sources. In this context, we also present a new source of data, highly correlated with the ILI rate. Saúde 24 is a Portuguese national triage call centre service, established to give free and real-time telephone health advice [21]. From the symptoms collected in each phone-call, we can not only have an estimate of the Portuguese ILI rate, but also use our method to signal the flu season outbreak.

**Fig 1:**
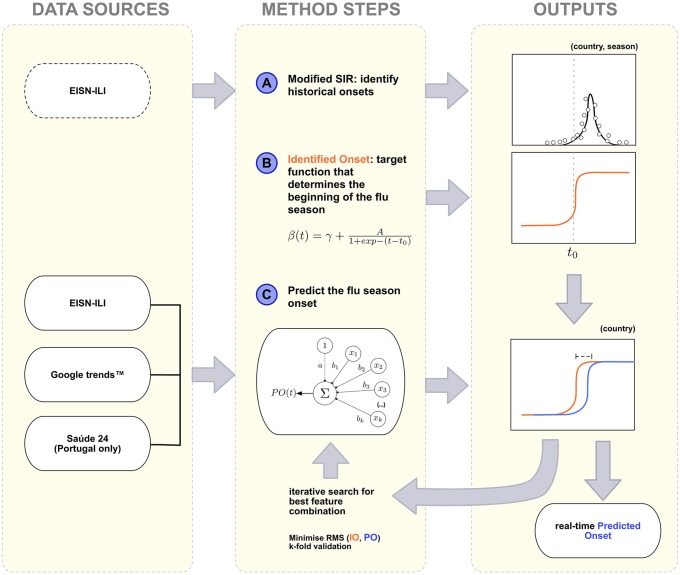
3-step flow diagram. Data Sources and diagrammed Method used in this study. Left column (labelled Data Sources) shows the different data used at the different stages; the middle column (labelled Methods Steps) shows the different methods and approaches developed; the column on the right (labelled Outputs) shows the fits and curves used for comparison. A) First step (top row, left to right): from the EISN-ILI data and by applying a Modified SIR model, the onset of five consecutive Influenza seasons was identified (represented by the dashed vertical line in the top plots, “Outputs” column, *t0*); B) Second step: an Identified Onset (IO) function is defined and centred at the previously found onset week (B and orange line on the “Outputs” column); C) Third Step: alternative ILI-related data sources (bottom left column) were used as input to create a Prediction Onset (PO) function (blue line, bottom right column), with the Identified Onset (orange line) function as the target output. The Predicted Onset is chosen in an iterative process, as the one that minimizes the difference to the Identified Onset. This function is fixed and then used for real-time prediction.

First we identify the onset of the flu season by fitting the EISN-ILI data using a modified version of the classic SIR model (MSIR), with a dynamic transmission rate (A and B in Fig 1). This marks the beginning of the flu in past seasons and in several countries. Second, we use alternative data sources, that do not have the described limitations of the EISN-ILI data, to identify the onset, using the MSRI fitted EISN-ILI as our ground truth (C in Fig 1). Our model selects the combination of features that minimizes the difference between the onset identified using the EISN-ILI fit (orange lines) and the one obtained with the different data sources (blue line). Third, we test this model in real-time and compare our predictions to the target. Finally, we compare our real-time identified onsets to the official flu season alerts, as published by the different countries analysed.

By using these different data sources, and optimizing each source’s strengths, we can produce an accurate signal that identifies, in real-time, the onset of the flu season and that anticipates the official alerts by several weeks. We show that the model performance depends not only on the quality of the input data, but also on its diversity. We also show that the model is not region-specific and that, depending on the quality of the data, can be applied to different countries.

With such a reliable method, complementary to the current system, public health authorities could significantly anticipate their respective protocols and timely respond to the upcoming flu peak.

## Materials and Methods

### Data

We collected influenza related data from three different and independent sources. These are the 1) EISN-ILI incidence rate per 100,000 inhabitants, considered the ground truth in this study, 2) Google Trends for four influenza related search-terms and 3) Saúde 24 phone calls logs, only available in Portugal. When possible, we collected this data in all countries under consideration and for five consecutive influenza seasons: 2010/2011, 2011/2012, 2012/2013, 2013/2014 and 2014/2015.

#### Data and country selection

Control data (for both training and method testing) was the weekly registered ILI incidence rate per 100,000 inhabitants for 23 European countries from June/2010 to June/2015. This data was provided directly by ECDC in the context of the European Influenza Surveillance Network (EISN) [22], in July 2015, and is referred to, throughout the text as EISN-ILI (Note: we asked for the data corresponding to the 29 countries in the database but we could could only collect it for 23 countries, for these seasons, please see S1 Table.)

**Fig 2:**
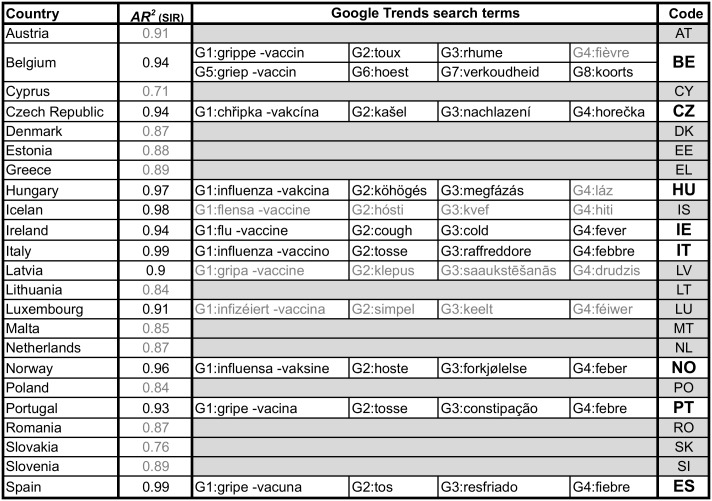
Countries analysed and GT search terms. First column shows the 23 countries for which EISN data was collected. Second column is the *Averaged Adjusted R^2^* resulted from the MSRI fit. Grey font represents countries that did not fulfil the two selection criteria (*AR*^2^ > 0.9 and convergent fit in all seasons) and were eliminated. Columns 3 to 6 show the GT terms searched for in each country. Grey font represents searches that did not have enough GT search volume. Countries that did not have at least three GT time series were eliminated, so that the final set includes countries with at least two independent sources of data. Last column shows the country codes used, with grey cells marking the countries that were discarded and white bold cells the eight countries that were further analysed in this study.

We applied a modified SIR model, explained in detail below, to each influenza time series and selected as case studies the countries for which the fits fulfilled two criteria: 1) all fits followed a SIR-like curve; and 2) the *Averaged Adjusted* *R*^2^, among all five seasons’ fit, was higher than 0.9, i.e., *AR*^2^ > 0.90. These selection criteria made it possible to further analyse eleven countries: Belgium (BE), Czech Republic (CZ), Hungary (HU), Iceland (IS), Ireland (IE), Italy (IT), Latvia (LV), Luxembourg (LU), Norway (NO), Portugal (PT) and Spain (ES). Fig 2 shows the considered countries (first column), the respective Country Code used in this paper (last column) and the quality of the fit, (*AR*^2^, second column—countries that did not fulfil the two criteria are grey).

#### Google Trends

As a second set of input data we retrieved the frequency by which people in different countries searched for influenza related terms on Google, from June 2010 to June 2015. Collection was performed on September 2015. Google Flu Trends (GFT), the discontinued Flu surveillance system developed by Google, was only available in select countries and was only retrieved for BE, ES, HU and NO. This data was obtained from https://www.google.org/flutrends/about/ and the time series broadly correspond to the ILI curves (data not shown). Instead, we used the freely and widely available Google Trends (GT) [23] and fixed four search terms in all selected countries: “flu -vaccine”, “cough”, “cold” and “fever” as these are present in all ILI definitions [24]. These were translated into the local language(s) using Google Translate [25] together with the respective Wikipedia [26] articles, and were then validated either by native speakers or by other sources. Fig 2, columns 3 to 6, show the search terms, and the corresponding time series are shown in S1 to S8 Figs.

GT time series depend on the total traffic search volume. Countries for which we could not retrieve at least three (out of the four influenza related terms), GT weekly query time series, during the analysed time period, were removed from the study. This was the case of Iceland, Latvia and Luxembourg, as well as the time series for the search terms “fièvre” and “láz” for Belgium and Hungary, respectively (marked in grey in Fig 2). The query search condition “-vaccine” removes the searches that included the word “vaccine” as these searches are more frequent at the beginning of the vaccination season and created an artificial peak.

#### Saúde 24 data

A third source of explanatory data was considered for the Portuguese case. Saúde 24 (S24) is an on-call service provided by the Portuguese National Health Services, available 24 hours a day, and established to triage for health conditions (symptoms and signs) and provide recommendation and counselling on the adequate level of care. Started in 2007, the services receive approximately 700.000 phone calls, annually. Based on the described symptoms, a trained S24 nurse follows a computer algorithm and selects a specific health protocol, which will then result in proper clinical advice. S9 Fig shows the total number of phone calls received by S24 services between June 2010 and February 2015, and the reported ILI incidence rate for Portugal in the same time period. No in-depth study of the S24 caller population has been made but this is a very accessible and almost cost-free service (local phone-call charge). S24 data is extracted from electronic registries of calls that use unique identifiers (NHS personal ID). The data has no information, recall, or misclassification bias. Each phone call is registered and labelled with date and time of call, age and gender of caller, location at the time of the call, and the respective health condition protocol. The nurse operators are also encouraged to fill-in a free text field, with comments that include the patient’s complaints, based on self-reported signs and symptoms.

From the 118 available health condition protocols and, following S24 expert advice, we listed all phone calls that activated one of the 15 protocols that could be caused by Influenza (see S2 Table). Data collection took place in March 2015. To gain explanatory power, we divided the caller’s age in four age groups: 0–4, 5–24, above 25 years old and created a fourth time series with all phone logs, regardless of age. Then, we extracted the patient reported symptoms directly from the free-text comments and searched for and counted occurrences of relevant words in each phone call (see S3 Table). A mention to any of the 11 words listed in the left column was sufficient to include the call in the time series and no further events would be counted: each call can correspond to one event only. These search terms were then grouped as shown in the same table so that 24 time series were generated. The obtained time series were normalized by absolute number of phone-calls to buffer the method from overall magnitude differences that might occur from season to season (e.g. due to varying media coverage or dissimilar S24 team size during the year). The right-size column of S3 Table shows each time series’ label and S10 Fig shows the corresponding data.

#### Geographic spread of influenza

To identify the official beginning of the flu season in each country we used the Geographic Spread of Influenza (GSI) reported weekly by FluNet [27] in the context of Global Influenza Surveillance and Response System (GISRS). This indicator is provided remotely by each country’s National Influenza Centres (NICs) and varies from country to country. Countries can inform on the dispersion state of the epidemic through five levels [28]: No activity, Sporadic, Local Outbreak, Regional Activity and Widespread activity. For the purposes of this work, we have considered the onset of the influenza season when Regional Activity is reached. Therefore, when we mention “alert period” we are referring to the time period that started when at least “Regional Activity” level has been declared. We repeated the analysis using the even more conservative threshold of Local Activity, but found that it did not change our comparison in a noteworthy manner, as it is a less common measure.

### Methods

The main goal of our approach is to build a mechanism able to timely identify the flu onset. Our method relies on a function that signals, in real-time, the likelihood that the season has started. This signal function receives as input influenza related data, and outputs a normalized sigmoid-like activation function that informs about the likelihood of the onset. To build such a signal we devised a 3-step method (Fig 1).

First, and in order to construct the training data sets, we identified the week that marks the beginning of each season, or onset. This is done by fitting a Susceptible-Infected-Recovered-like compartmental model to the EISN-ILI data of all influenza seasons under consideration.

Second, we introduce a signal function, the *Identified Onset* (IO), centred at the previously found onset week. This is used as the ground truth or target function, to which all other simulations will be compared.

Third, by using alternative data sources, a training process is explored to fit a *Predicted Onset* (PO) signal function by repeating and testing over all seasons.

Finally, we compare both the Identified and the Predicted Onsets to the official alert periods, described in the Data section. The following sections describe each step in detail. We have used Mathematica 10.1 [29] to perform all calculations

#### Identifying the onset

Identification of the onset of the flu season is not trivial and onset determination varies from country to country. However, current traditional methods usually define a baseline for the number of cases, from previous seasons, which needs to be crossed. In addition, it is also common practice to send some samples for laboratory testing, requiring one or two weeks of consecutive positive influenza results to release public alerts. This results in an overall slow mechanism. Our method relies on an automatic identification system, that does not depend on the absolute number of cases (the “previous-seasons baseline”). To “mark” the actual week of the influenza season onset, we devised modified version of the classical Susceptible-Infected-Recovered (SIR) model [30], where instead of the usual constant with time transmission rate, *β*, we used a time-dependent transmission rate with a sigmoidal shape. Thus, our method relies on a baseline calculated from the current season and detects significant changes from it. We will refer to this model as MSIR and it is based in the differential system of equations shown in Eq 1:

$$\begin{array}{cc} \frac{dS}{dt} & =-\frac{\beta SI}{N}\\ \frac{dI}{dt} & =\frac{\beta SI}{N}-\gamma I\\ \frac{dR}{dt} & =\gamma I\\ \beta (t) & =\gamma +\frac{A}{1+\exp-(t-t_{0})}\\ i_{0} & =I(t=0) \end{array}$$ (1)

where *β* and *γ* are the transmission and recovery rates, respectively; *S*, *I* and *R* the susceptible, infected and recovered compartment sizes, respectively; *N* is the population size (set to *N* = 1000); *i*_0_ is the initial condition that, together with *A* and *t*_0_, is adjusted during the fitting process. Additionally, because we are only interested in defining the onset week (and not in any other common epidemiological measures, such as the amplitude of the peak), we use the rescaled ILI rate with the season peak set to 100. Using the same scale for all seasons prevents the parameter fine-tuning search intervals, that would be needed for each season and for each country. To test whether this rescaling has any impact on the sensitivity of our method, we have fitted the Portuguese ILI records of all 5 seasons with and without rescaling and it resulted in an onset location with no significant differences (less than two days).

We used a non-linear model fit together with a parametric search, to find the best numerical solution of the ordinary differential system of equations shown in Eq 1. The parametric search interval was fixed with 0.1 < *γ* < 5, 0.1 < *A* < 5 and 0.1 < *i*_0_ < 20. We evaluated the quality of the fit using Least Square (LS) analysis and considered the fit to be good when the *Averaged* *AR*^2^ among all five seasons’ fit, was higher than 0.9. This method was chosen for two main reasons: first, at this stage we are interested to find the best overall data description through the modified SIR, and LS offered the best quality fits (essential for the quality of the onset determination); second, due to its simplicity and common practice. We have also tested an alternative fitting approach, through a Maximum likelihood estimation assuming a Poisson distribution. This approach did not grossly alter our average onset determination (average change for all countries and seasons under consideration was close to one week) but it resulted in poorer data adjustment.

The dynamics of the system of Eq 1 have two periods. When *t* <<*t*_0_, *β* ≃ *γ*, or the basic reproduction number, *R*_0_ = 1, and the epidemic has not yet started. When *t* >>*t*_0_, the rate of new infections exceeds the recovery rate, which results in a epidemic situation. Therefore, the onset week marks this transition, identified as *t* = *t*_0_. As we show below, the fitted time mark *t* = *t*_0_ is always located at the inflection of the SIR-shaped growth curve, which makes it an excellent candidate to define the onset week.

#### Signal functions

After identifying the onset, we built a signal function that outputs the likelihood that this onset has been triggered. It is designed to be equivalent to the transmission rate described above, but normalized and centred at the onset time. This “Identified Onset” (IO) function is shown in Eq 2, with *t*_0_ corresponding to the MSIR fit onset week, the point at which *I*_0_ = 0.5 (the inflection point). This signal function is the target function, used in the forthcoming training process, and therefore plays the role of the **true** underlying model.

$$\begin{array}{c} IO^{i}\left(t\right)=\frac{1}{1+\exp[-\left(t-t_{0}\right)]}\;i=season \end{array}$$ (2)

In order to map the input features, i.e., ILI, Google Trends volume searches and/or Saúde 24 data, to the target “Identified Onset”, we devised a simple sigmoid activation function, the “Predicted Onset” (PO) signal function, shown in Eq 3:

$$\begin{array}{c} PO^{i}\left(t\right)=\frac{1}{1+\exp[a+\sum_{k}b_{k}x_{k}\left(t\right)]}\;i=season \end{array}$$ (3)

where *x*_*k*_(*t*) are the features at time t, and the set {*a*, *b*_*k*_} are fitted weighting parameters. Therefore, the training process finds the set {*a*, *b*_*k*_} that best reproduces Eq 2, i.e. the set for which the Predicted Onset is closer to the Identified Onset.

To perform this calculation in real-time, two data processing steps were necessary, that do not interfere with the conclusions. First, and as we consider the pre-peak scenario only, the time series were truncated to the maximum registered ILI record of the respective influenza season. Second, we applied a smoothing algorithm to all time series: starting from the first two recorded weeks, a linear fitting process was calculated and the process was repeated for every subsequent point, as the data became available.

#### Training and proposed onset

To gain generalization and predicting power for upcoming seasons, we applied the training process to all five seasons, using a k-fold cross validation (CV) approach, with *k* = 5 [31]. Each country’s dataset was divided in five sub-datasets. Each sub-dataset included one season as a test set and the remaining four as a training set, so that all five seasons were used as both training and test sets. For each training set we performed a non-linear fit of Eq 3, with *IO*^*i*^(*t*) as our target function. The quality of the fit was assessed using the *averaged root mean square* (RMS) of the simulated test season.

The simultaneous inclusion of all possible input time series (EISN-ILI, GT and S24) for the respective country, does not necessary result in the best fitting scenario, as the fitting optimization process may encounter several local minima. Therefore we searched for the combination of time series that offered the best predictive power, in three steps: first, using the averaged RMS from EISN-ILI data only (using the EISN-ILI record from the previous week as *PO*^*i*^ input (*ILI*_−1_)); second, seeking the best combination of variables using as input the GT set alone; third, repeating the search considering *ILI*_−1_ + *GT*. We proceed in the same way for the Portuguese case, having added an individualized search from the S24 set.

**Fig 3:**
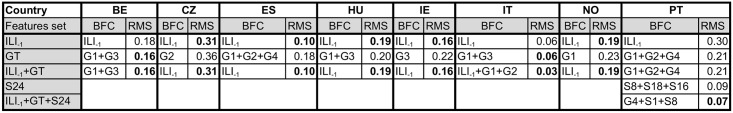
Best feature combination and corresponding averaged root mean square (RMS). RMS when only the ILI time series were considered (third row), when only GT time series were considered (fourth row), when both ILI and GT were combined (fifth row), when only S24 time series were considered (sixth row), and when all features were combined (Portugal only, seventh row). The minimum RMS for each country is shown in bold and the corresponding feature combination is considered the best (BFC).

For the countries with less than 5 input time series, we performed the calculation for all possible combinations. This was the case of Czech Republic, Spain, Hungary, Ireland, Italy and Norway. In the cases of Belgium and Portugal, for which the large number of time series rendered it impossible to test all combinations, we implemented a search algorithm for the best input set, which works as follows: first, we applied the fitting process to each individually possible input time series, separately. Second, and from the previous result, we selected the time series that minimized the averaged RMS. Third, we looped through all other time series, combined with the previously selected one(s). If a new combination improved the RMS, the new time series was included and the process was repeated until no averaged RMS improvement was observed. Fig 3 shows the best combination of features found for the different countries, and the corresponding RMSs

## Results

### MSIR

**Fig 4:**
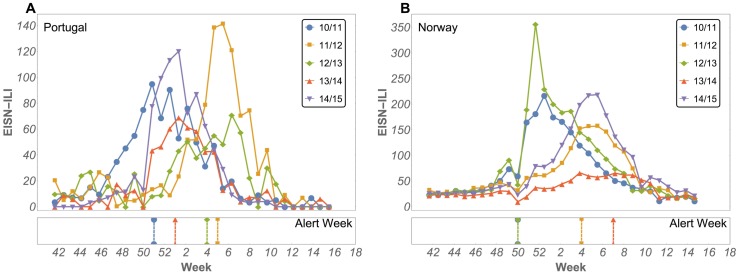
ILI rates. ILI rate per 100k inhabitants (top panels) and week of the official onset alert (bottom panels) for Portugal (A) and Norway (B) for five consecutive seasons 2010–2014, according to the EISN. No alert report for the 2014/2015 season was available at the time of data collection.

The influenza epidemic varies in timing and amplitude from season to season and from country to country, making it difficult to predict and identify its onset. Fig 4 reveals this difficulty by showing, for two geographically distinct countries, the unscaled ILI incidence, as obtained from the EISN, and comparing five seasons with the official alert dates. S11 Fig shows the same data for the other countries analysed. To help identify the onset, we applied the modified SIR model (MSIR) to all 23 countries available from EISN. From these, 11 countries resulted in a good fit (all 5 seasons fits must result in a SIR shape distribution and *AR*^2^ > 0.9), and for 8 countries we had at least two independent data sources. Fig 2 shows the countries analysed and the time series used as inputs (columns 3 to 8). These countries have different climates, cover a large geographical area and have significant cultural and social differences, from language to school year.

**Fig 5:**
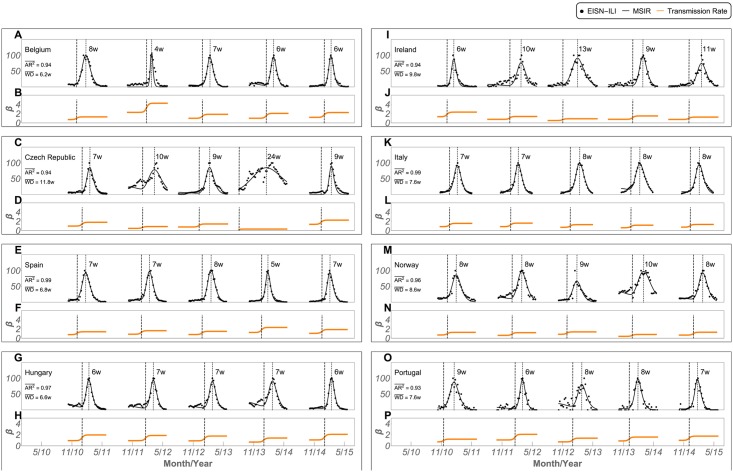
Best MSIR fitted results in converging countries, from 2010–2015. Panels A, C, E, G, I, K, M, and O show the best MSIR fitting results and averaged *AR*^2^, where a rescaled EISN-ILI rate with the season peak set to 100 was used. Panels B, D, F, H, J, L, N, and P show the corresponding transmission rate (*β*). Dashed vertical lines connecting both panels show the transmission rate inflection point. The week difference (WD) between the infection point and the maximum MSIR fit is shown for each season and its average shown on the left of the top panels.

Fig 5 panels A, C, E, G, I, K, M, and O show the best Modified-SIR model(MSIR) fitting results for seasons 2010–2015, for each of the analysed 8 countries (we show in S12 Fig the equivalent results for Iceland, Luxembourg and Latvia). The corresponding best fit parameters are summarized in S4 Table. Each fit is accompanied by the respective transmission rate, *β*, obtained from Eq 1 (orange line in Fig 5 panels B, D, F, H, J, L, N, and P). The dashed vertical lines, connecting both panels, show the transmission rate sigmoidal inflection point, which marks the epidemic onset. We show also in S13 Fig the fitting result for all initial analysed 23 countries.

As can be seen, there are large differences in amplitude and smoothness between (and within) seasons, but as long as our MSIR fits the EISN-ILI time series, we can identify the beginning of the season as the inflection point of the sigmoid (dashed line) in all 8 countries.

Given it’s sigmoidal shape, the transmission rate is a strong candidate indicator of the epidemic onset: it results in an approximated step function that triggers at the outbreak. Thus, we can define “signal functions”, with a similar shape, and collect alternative sources of data, that do not require post-processing, such as the one provided by Google Trends (GT). By using a combination of time-series and by training our functions, we can identify the onset in real-time. The figure also shows the week difference between our identified onset and the MSIR fit maximum value, which can be interpreted as the time difference between the beginning of the season and its peak. This week difference is consistent in several countries (Hungary and Spain being the best examples), with an overall average (and median) distance of 8 weeks.

### Input Data Sets—Features

S1 to S8 Figs show each GT query time series, for the respective country, and the corresponding EISN-ILI rate, between June/2010 and June/2015. As mentioned before, GT does not release weekly data for every search term, in every country. Fig 2 shows the countries for which only monthly data was available and notes whether they were removed from the study.

Similar to previous work in other countries [7], we find a good peak correspondence between the EISN-ILI incidence and searches for influenza related terms, particularly for the words “flu” and “cough”, in the different countries and languages analysed. However, there have been some concerns regarding the use of GT to predict and track the flu season [32][33]. These are mainly focused on its sensitivity to media reporting, which can lead to an artificial increase in searches, and the covered demographics, which in some countries is heavily biased towards a young and educated population. To overcome these issues, we also took advantage of the Saúde24 (S24) phone service, which covers a broader demographic, including elders, also offering detailed information about the callers. These calls are in real-time and the call logs provide both structured and unstructured information about the callers’ symptoms as gathered by highly trained nurse practitioners. Moreover, S24 became well known in Portugal during the 2009 H1N1 pandemic and it is still broadly used by people with ILI symptoms. S9 Fig shows the total number of phone calls received by S24 services between June/2010 and February/2015, and the reported EISN-ILI incidence rate for Portugal in the same time period. Similarly to GT, we find a good correspondence between the number of calls that S24 received and the EISN-ILI incidence.

### Best Feature Set Combination

We then combined these different input time series, or features, to determine which combination offered the best onset identification (see Methods for more details). These are the input combinations that minimize the difference between our Identified Onset (IO) and our Predicted Onset (PO), and are shown in Fig 3. S5 Table shows, for these best selected features, the resulted weights set {*a*, *b*_*k*_}.

In half of the countries, using ILI provided best fits than using GT (CZ, ES, IE and NO). The reverse was true in Belgium and Portugal, with no noticeable differences in the cases of Hungary and Italy. Using a combination of features, either from ILI, from different GT term-searches, or S24, proved to be the best option for Italy and Portugal. This consistency between the IO and the PO was particularly good in the cases of Italy (RSM = 0.06) and Spain (RMS = 0.11), which were also the countries that had presented the best *AR*^2^ in the MSIR fit (Fig 5). This is not a coincidence, as well-shaped data clearly offers the best prediction results.

In the case of Portugal, that did not have a particularly good *AR*^2^, adding the features from S24 alone, improved the signal by 2 to 3-fold when compared with the other features’ results and by 2-fold when compared with the average best RSM of all the other countries that, to our knowledge, do not have an S24 equivalent.

### Signal Prediction Simulation

**Fig 6:**
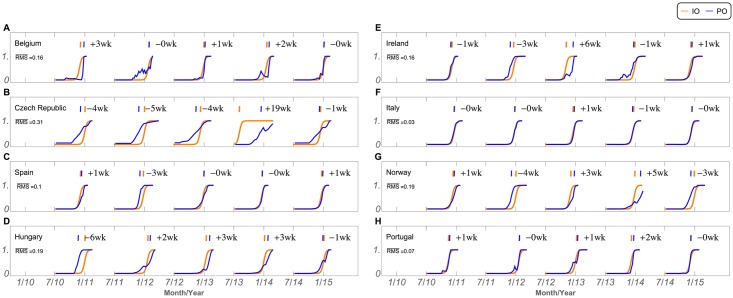
Onset detection. Identified Onset (IO, orange line) and Predicted Onset (PO, blue line), for all seasons (x-axis) and countries (panels A to H). Onsets are identified at the inflection points of the curves, at 0.5. Averaged RMS between the IO and PO is shown at the left. Week differences between the inflection points of IO and PO are shown at the top, by season. IO corresponds to the normalized transmission rate *β* shown in Fig 5.

The previous results show that it is possible to select different features and to use them to identify the onset of the flu season, in different countries. We then trained our model to simulate a real-time signal prediction, using the previously selected features. Fig 6 shows the signal prediction simulation for 2010–2015 seasons, and for the different countries. It is possible to see the predicted onset (PO) function, in blue, and the identified onset (IO) function, in orange, for all five seasons and for all 8 countries. The onsets are chosen as being the curve’s inflection points, at 0.5.

The prediction simulations were very good for Spain, Italy and Portugal, followed by Hungary, which presented a sigmoid shape and a stable signal. Again, the best simulation cases agreed with the best MSIR fitting scenarios (Spain and Italy resulted in a *Adjusted* *R*^2^ of 0.99 and 0.97 respectively) or, in the case of Portugal, when the S24 data was included. It should also be noted that, except in a few cases discussed below, all calculated feature weights are within the range of the standard error, for the respective season and country (S5 Table). This is a good indication that our results are not over-fitted.

The same figure also shows the week difference between the orange IO and the blue PO trigger week position. A plus signal means that the prediction failed by lateness, and the minus sign that it failed by anticipation. We found a close to perfect match (0 and plus or minus one week) in approximately half of the seasons analysed. The model misses the onset by 4 or more weeks in only 8 out of the 40 season/country combinations, most notably in the cases of Czech Republic, Ireland and Norway, with 4 of these instances being a signal anticipation, or false positive. In fact, one of the few exceptions to the quality of the feature weighs is found in season 10/11, in Hungary. In this case, the resulted fitted *a* constant (S5 Table) is very far from the other comparable four results, most likely a consequence of the fitting optimization process. Thus, the result is a poor generalization for the season prediction simulation and, in this particular case, it resulted in a 6 week false positive. However, it is worthwhile noticing that this might not be a real failure of the model. S4A and S11D Figs, show that there is a systematic (and most likely artificial) decrease in the registered number of cases every season on, and around, week 50, in Hungary. Our method identified week 48 as the beginning of the season 10/11 and it is possible that this was actually the case.

**Fig 7:**
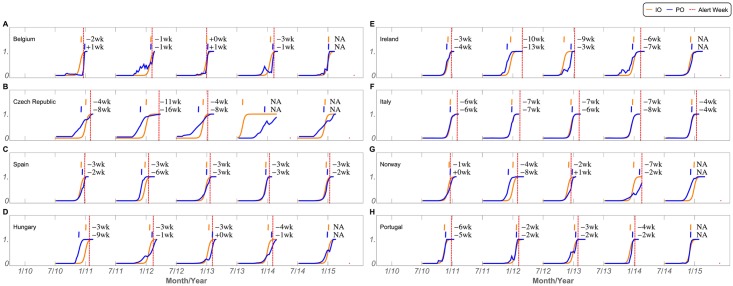
Onset anticipation. Identified Onset (IO, orange line), Predicted Onset (PO, blue line), and official alert week (Alert, dashed red line) for all seasons (x-axis) and countries (panels A to H). Onsets are identified at the inflection points of the curves, at 0.5. Week differences between IO, PO and the Alert are shown at the top, by season, in orange and blue, respectively. A minus sign means that the model anticipated the official alert and a plus sign means that the model was delayed in comparison with the official alert. NA means that at the time of collection, no official alert was available.

S11 Fig also shows that often the official alerts happen at or even past the peak. To compare our method to the timings of the current official alert system, we plotted the same IO and PO curves and added the official season alerts, as shown in Fig 7 and separately in S14 and S15 Figs, respectively. The IO matches or anticipates the official alert in all of the cases studied and anticipates the alert signal by at least 2 weeks in 90% of the cases (S14 Fig). In the case of Spain, these were very consistent, and the IO anticipated the alert by exactly three weeks in all seasons analysed. Similarly, the PO calculated by our real-time prediction, anticipates or closely matches (at most one week difference) the official alert in all but three of the seasons (BE 10/11, BE 12/13 and NO 12/13), and anticipates the alert by at least 2 weeks in 70% of the cases. These differences are particularly large in Czech Republic or Ireland, but consistent in countries such as Spain, Italy and Portugal, where we observe systematic predictions. This result would be even more striking if we removed Belgium from the analysis. In the case of Belgium, the correspondence between the PO and the Alert is very good, suggesting that the official alerts in Belgium happen with a very short delay. This clearly indicates that the official alerts are systematically delayed by at least a few weeks when compared to the actual beginning of the epidemic and that our method can improve the current system by more than 3 weeks, in most cases.

## Discussion

We have combined a modified SIR compartmental model, a proposed signal function and new sources of data to show that it is possible to: 1) use the inflexion point of a sigmoidal transmission rate as an indicator of the onset of the influenza epidemic; 2) identify this onset in real-time; 3) anticipate the current season alert by several weeks and 4) apply this method to different European countries.

Both EISN-ILI and GT work as very good input datasets, and we can identify features with good predictive power (*RMS* < 0.2, Fig 3) in all countries but the Czech Republic, for which the initial MSRI fit was already difficult. However, it is clear that no single input dataset (ILI, GT or S24 alone) offers the best results in all countries. For instance, including the GT time series improves the quality of the detection in Belgium, Italy, and Portugal, but not in the Czech Republic, Spain or Ireland. This is likely a reflection of the quality of the data. In some countries, there is a high variance of ILI records, especially for lower values and particularly common in the pre-epidemic period (see Fig 4 and S11 Fig). Conversely, the search volume for flu-related symptoms on GT is more common and consistent in some countries than in others. This makes a case for country specific analysis, instead of one-size fits all models. Indeed, flu surveillance seems to be an excellent example of a monitoring system where a combination of different datasets, computational analysis, and human input can be most valuable. And that our method is flexible enough to be used with different data series, independently or in combination, argues for its use as a tool that can be adapted to different countries and circumstances.

We also note that the PO function proposed in Eq 3 can be interpreted as a single neural activation function. Machine learning (ML) algorithms have recently been applied to many different problems, many of which require predicting a target function from a set of input numerical features. Finding the onset of the flu season, as well as predicting its dynamics, should be no exception. Our framework, and presented results, make a strong case for the application of ML techniques to the prediction of seasonal diseases. Specially, and as we show here, since it is possible to gather different sources of influenza related data, that can be used as explanatory input variables, and using more complex neural networks or decision trees might prove very powerful. A similar approach was very recently described in [9], and applied to USA data and focusing on the epidemic dynamics. Despite not focusing on onset prediction, this work supports the use of different datasets and ML methods to improve the current epidemic control.

In fact, that our simple GT dataset showed to be such a good tool was somewhat surprising, as there has been some controversy regarding the use of Google Flu Trends (GFT) as an accurate method to track the flu season [33][7]. We share many of the raised concerns, particularly GFT sensitivity to media reporting and variation in search profiles [33]. However, this is more likely to happen after the season has been announced and the population is already showing ILI symptoms. Since our method only focuses on the early stages of the epidemic, it might be less subject to media or pre-emptive searches. On the other hand, when the search volumes are low, it might not have enough power to detect the initial variations. This limitations could be partially circumvented if Google allowed access to the raw absolute number of searches, instead of the normalized and varying version that GT currently offers. And using GFT instead of the more general GT could possibly improve the results even further. However, [33] analyses three different USA regions to show that the 2013 updated GFT algorithm misses the onset by at least 2 weeks in 17 out of the 29 seasons (close to 60%) for which they could compare. Searches on Google are likely to vary from country to country, partially explaining its varying success, but it is also possible that the GFT algorithm itself offers poor results, as had already been suggested [32]. Moreover, this service is only available in select countries and its algorithm is proprietary, making it difficult to use. Thus, and despite the described limitations, we argue that even if GT might be less than optimal to predict the peak, it does prove to be very useful in detecting the onset of the seasonal influenza epidemic, in most countries.

In addition, we have presented a new and potentially very useful tool, S24, to do early detection of epidemics. It does not have many of the disadvantages of previously used input methods, as it covers the broader demographics of people who have access to telephones, there is trained medical personnel on the other side of the phone line, prepared to distinguish between real symptoms and unfounded concern, and the entire system works in real-time, ready to direct (or deflect) callers to (from) emergency rooms. Similar systems are being implemented in several countries [34][35] and we expect it to become a great asset in real-time detection and health management.

### Limitations

The proposed model is not designed to detect the peak: there is no focus on the amplitude of the curve as it depends on external factors that were not considered. This can also explain why the search engine Google has shown to be an accurate tool. The model is also not designed to detect off-season events, such as the 2009 pandemic, as it requires defining a baseline from which the onset deviates. Finally, the fact that the method works differently in different countries can be described as both an asset and a limitation. Our model is flexible enough to be generalized to many countries and realities, contrary to the majority of the previous work that has been limited to the USA and other specific countries. However, it requires fine-tuning to each countries’ data sources and requires that the seasonal epidemics follow a well-behaved SIR-like curve.

### Conclusion

Overall, the system that we have developed, due to its accuracy and simplicity, by providing one single, easy to interpret, output, can be very useful for public health authorities, in tracking and identifying the beginning of the flu season. In fact, our system is currently being tested in real-time, together with the relevant Portuguese health organizations, and should be easy to implement in other countries. Moreover, and also due to both its simplicity and to the fact that it can be used with different input data, this method should be easy to apply to other SIR-like contagious or seasonal diseases.

## Supporting Information

S1 Fig ILI rates and GT search terms. ILI rates per 100k inhabitants (A) and GT volume search (Vol.) for 7 different search terms (B to H) for Belgium for five consecutive seasons 2010–2014. In the case of Belgium the search terms were translated to both French and Flemish. The word “fièvre” had not significant search volume and was not included. GT time series normalized by Google, and the maximum search volume for each term is set to 100.
(TIF)

S2 Fig ILI rates and GT search terms. ILI rates per 100k inhabitants (A) and GT volume search (Vol.) for 4 different search terms (B to E) for Czech Republic for five consecutive seasons 2010–2014. GT time series normalized by Google, and the maximum search volume for each term is set to 100.
(TIF)

S3 Fig ILI rates and GT search terms. ILI rates per 100k inhabitants (A) and GT volume search (Vol.) for 4 different search terms (B to E) for Spain for five consecutive seasons 2010–2014. GT time series normalized by Google, and the maximum search volume for each term is set to 100.
(TIF)

S4 Fig ILI rates and GT search terms. ILI rates per 100k inhabitants (A) and GT volume search (Vol.) for 3 different search terms (B to E) for Hungary for five consecutive seasons 2010–2014. The word “láz” had no significant search volume and was not included. GT time series normalized by Google, and the maximum search volume for each term is set to 100.
(TIF)

S5 Fig ILI rates and GT search terms. ILI rates per 100k inhabitants (A) and GT volume search (Vol.) for 4 different search terms (B to E) for Ireland for five consecutive seasons 2010–2014. GT time series normalized by Google, and the maximum search volume for each term is set to 100.
(TIF)

S6 Fig ILI rates and GT search terms. ILI rates per 100k inhabitants (A) and GT volume search (Vol.) for 4 different search terms (B to E) for Italy for five consecutive seasons 2010–2014. GT time series normalized by Google, and the maximum search volume for each term is set to 100.
(TIF)

S7 Fig ILI rates and GT search terms. ILI rates per 100k inhabitants (A) and GT volume search (Vol.) for 4 different search terms (B to E) for Norway for five consecutive seasons 2010–2014. GT time series normalized by Google, and the maximum search volume for each term is set to 100.
(TIF)

S8 Fig ILI rates and GT search terms. ILI rates per 100k inhabitants (A) and GT volume search (Vol.) for 4 different search terms (B to E) for Portugal for five consecutive seasons 2010–2014. GT time series normalized by Google, and the maximum search volume for each term is set to 100.
(TIF)

S9 Fig ILI rate and Saúde 24. ILI rates per 100k inhabitants (A) for Portugal and absolute number of phone calls received by S24 services (B) between June/2010 and February/2015.
(TIF)

S10 Fig Time Series of S24 calls. Percentage of occurrences (number of calls) of each term or related term (see S3 Table) in respect to the total number of calls received in the same considered period (one full week).
(TIF)

S11 Fig ILI rates. ILI rates per 100k inhabitants (top panels) for Belgium (A), Czech Republic (B), Spain (C), Hungary (D), Ireland (IE) and Italy (F), for five consecutive seasons 2010–2014. When available, the corresponding week of the official alert report is shown in the bottom panel.
(TIF)

S12 Fig Best MSIR fitted results for Iceland, Luxembourg and Latvia, from 2010–2015. Panels A, C and E show the best MSIR fitting results and averaged *AR*^2^, where a rescaled ILI rate with the season peak set to 100 was used. Panels B, D, and F show the corresponding transmission rate (*β*). Dashed vertical lines connecting both panels show the transmission rate inflection point. The week difference (WD) between the infection point and the maximum MSIR fit is shown for each season and its average shown on the left of the top panels.
(TIF)

S13 Fig Best MSIR fitted results. The fitting curves and averaged *AR*^2^ for all 23 countries for which we could collect EISN consistent data (see S1 Table). On the left, show the best MSIR fitting results and averaged *AR*^2^, where a rescaled ILI rate with the season peak set to 100 was used.
(TIF)

S14 Fig Alert anticipation. Identified Onset (IO, orange line) and official alert period (red shade) for all seasons (x-axis) and countries (panels A to H). Timing of the IO and the Alert are shown at the top, by season, in orange and red, respectively, with week differences in black. A minus sign means that the IO anticipated the Alert and a plus sign means that the IO was delayed in comparison with the official alert. NA means that at the time of collection, no official alert was available.
(TIF)

S15 Fig Alert anticipation. Predicted Onset (PO, blue line) and the official alert period (red shade) for all seasons (x-axis) and countries (panels A to H). Timing of the PO and the Alert are shown at the top, by season, in blue and red, respectively, with week differences in black. A minus sign means that the PO anticipated the Alert and a plus sign means that the PO was delayed in comparison with the official alert. NA means that at the time of collection, no official alert was available.
(TIF)

S1 Table ECDC data. Influenza data was requested from the EISN [22] on 23/06/2015, from the 2010/2011 to the 2014/2015 seasons, for all listed 29 countries. Google Trends (GT) data was retrieved on 3/09/2015 from [23]. The table shows (from column 1 to column 7): countries’ names; official country codes; data range of data received from the EISN; whether the data was “complete” (5 full seasons), “incomplete” (less than 5 seasons) or “inconsistent” (in the case of the UK the same week could have 1, 2 or 3 entries); the Averaged *AR*^2^ of the MSIR fit; how many seasons presented convergent fits; for how many Google Trends search terms we could obtain time series; whether the country data fulfilled all the inclusion criteria. Countries were not considered in the analysis if we could not collect 5 consecutive flu seasons (eliminating BG, HR, FR, DE, SE and UK), if the Averaged *AR*2 < 0.9 or if not all 5/5 seasons showed a convergent SIR-like shaped fit (eliminating AT, CY, DK, EE, EL, LT, MT, NL, PL, RO, SK and SI), and if at least 3/4 of the tested GT search-terms had enough search-volume to generate a time-series (eliminating IS, LV and LU). Criteria that were not full-filled are marked as grey cells. Only the countries for which the entire row is white (also bolded) were accepted. These are BE, CZ, HU, IE, IT, NO, PT and ES.
(TIF)

S2 Table Selected Saúde 24 health protocols. Time Series of S24 calls. Occurrences of each term or related term (see S3 Table) were counted and plotted as time series, with one call corresponding to only one event. The caller’s age was divided in four age groups: 0–4 (labeled 4yrs), 5–24 (labeled 25yrs), above 25 years (labeled >25yrs) and a fourth time series with all phone logs, regardless of age (labeled)” years. The boxes on the right show the time series names, according to S3 Table.
(TIF)

S3 Table Saúde 24 time series. From the chosen protocols (shown in S2 Table) all phone calls that included at least one of the eleven words shown in the first column were selected. These words were grouped into six “general ILI symptoms”, in bold. These were further divided into four age groups, giving rise to twenty four time series (second column), numbered as shown in the third column.
(TIF)

S4 Table MSIR resulted best fit parameters. Best fit parameters resulted from the MSIR non-linear fit applied to the respective ILI rates.
(TIF)

S5 Table Best fit resulted weights. Best fitted {*a*, *b*_*k*_} set for Belgium, Czech Republic, Spain, Hungary, Ireland, Italy, Norway and Portugal, for five consecutive seasons 2010–2014.
(TIF)

S1 Data Official alerts data file. Official alerts time series for Belgium, Czech Republic, Spain, Hungary, Ireland, Italy, Norway and Portugal, for five consecutive seasons 2010–2014. Missing data was set as −0.1 [27].
(XLSX)

S2 Data Google Trends data file. Google Trends search volumes time series for Belgium, Czech Republic, Spain, Hungary, Ireland, Italy, Norway and Portugal, for five consecutive seasons 2010–2014 [23].
(ZIP)

S3 Data Saúde 24 flu records data file. Number of phone calls reported with flu symptoms time series.
(CSV)

S4 Data Saúde 24 total phone calls data file. Total number of received and answered calls.
(CSV)
