# Supplementary figures and images for: Early and Real-Time Detection of Seasonal Influenza Onset
Source: PLoS Comput Biol. 2017 Feb 3;13(2):e1005330. doi: 10.1371/journal.pcbi.1005330 (PMC5291378; doi:10.1371/journal.pcbi.1005330)

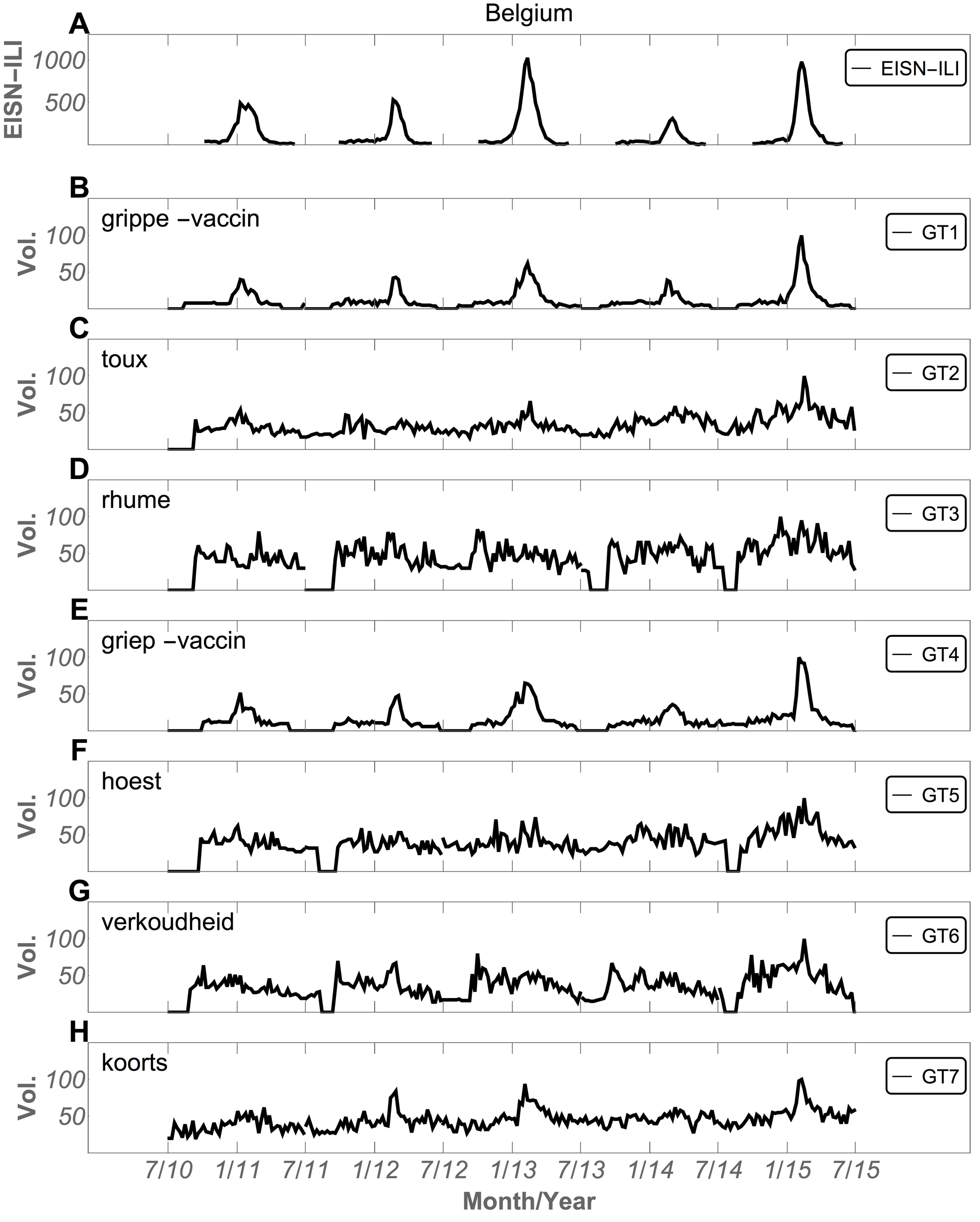

Supplement: S1 Fig — ILI rates per 100k inhabitants (A) and GT volume search (Vol.) for 7 different search terms (B to H) for Belgium for five consecutive seasons 2010–2014. In the case of Belgium the search terms were translated to both French and Flemish. The word “fièvre” had not significant search volume and was not included. GT time series normalized by Google, and the maximum search volume for each term is set to 100. (TIF) [file pcbi.1005330.s001.tif]

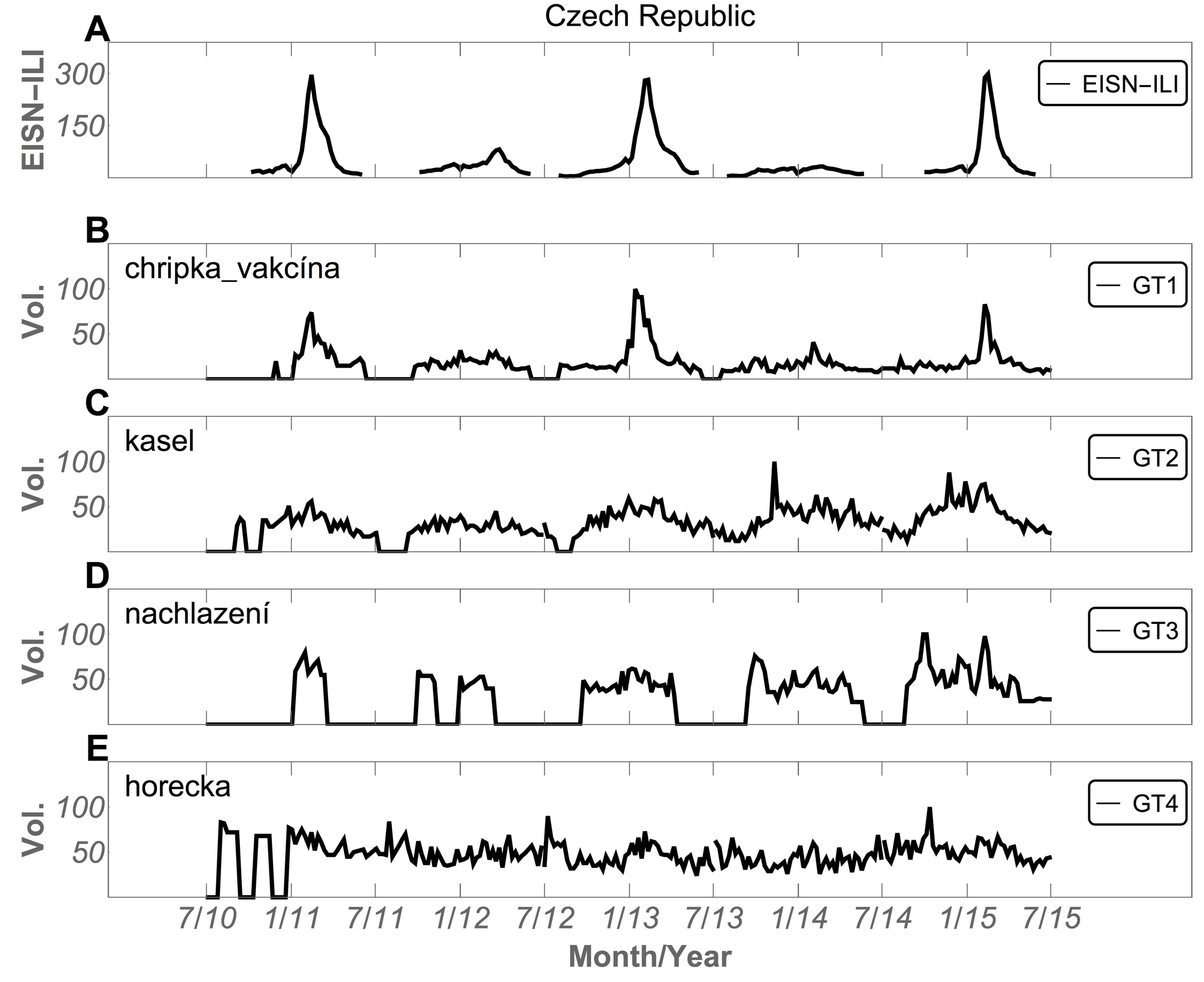

Supplement: S2 Fig — ILI rates per 100k inhabitants (A) and GT volume search (Vol.) for 4 different search terms (B to E) for Czech Republic for five consecutive seasons 2010–2014. GT time series normalized by Google, and the maximum search volume for each term is set to 100. (TIF) [file pcbi.1005330.s002.tif]

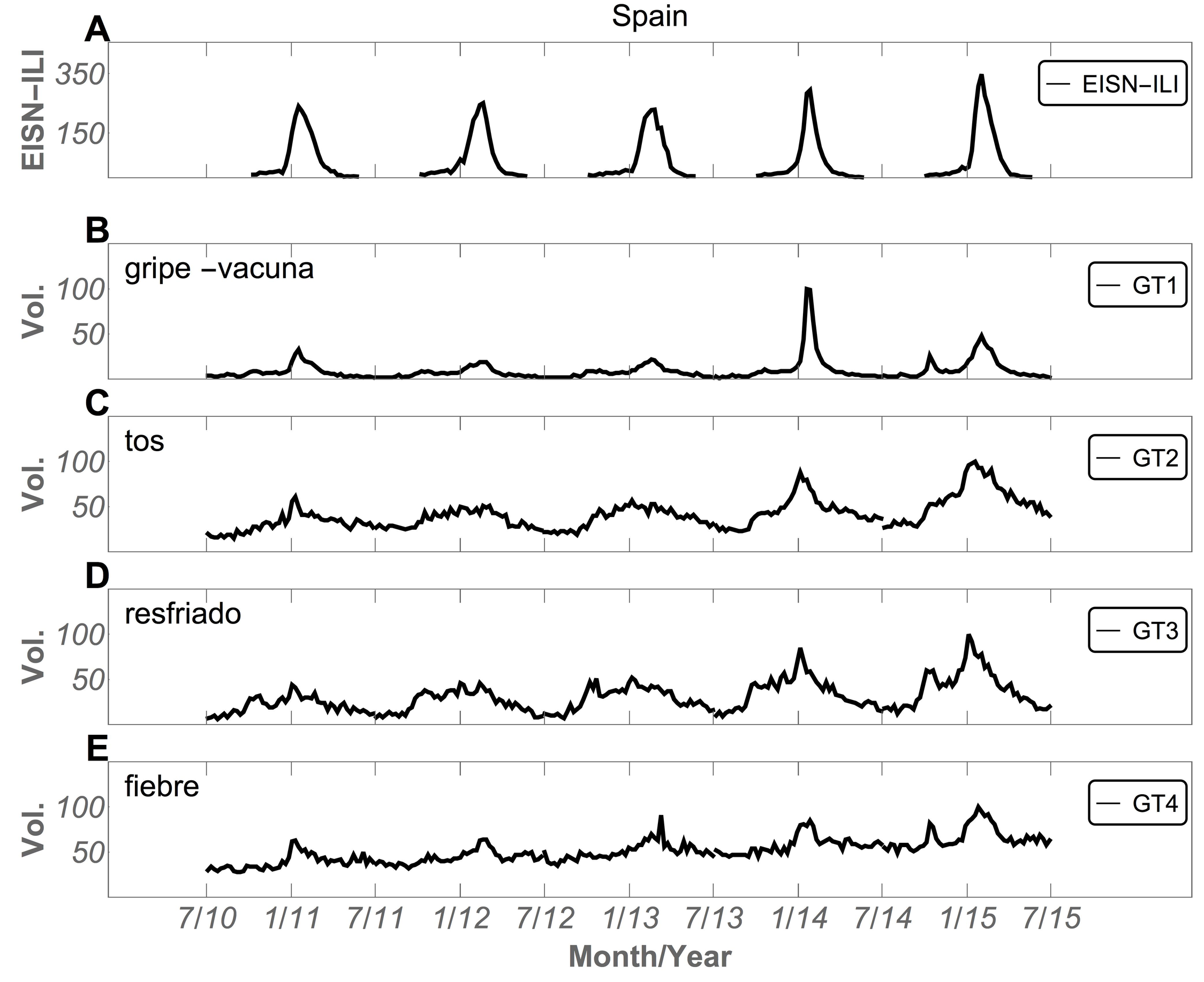

Supplement: S3 Fig — ILI rates per 100k inhabitants (A) and GT volume search (Vol.) for 4 different search terms (B to E) for Spain for five consecutive seasons 2010–2014. GT time series normalized by Google, and the maximum search volume for each term is set to 100. (TIF) [file pcbi.1005330.s003.tif]

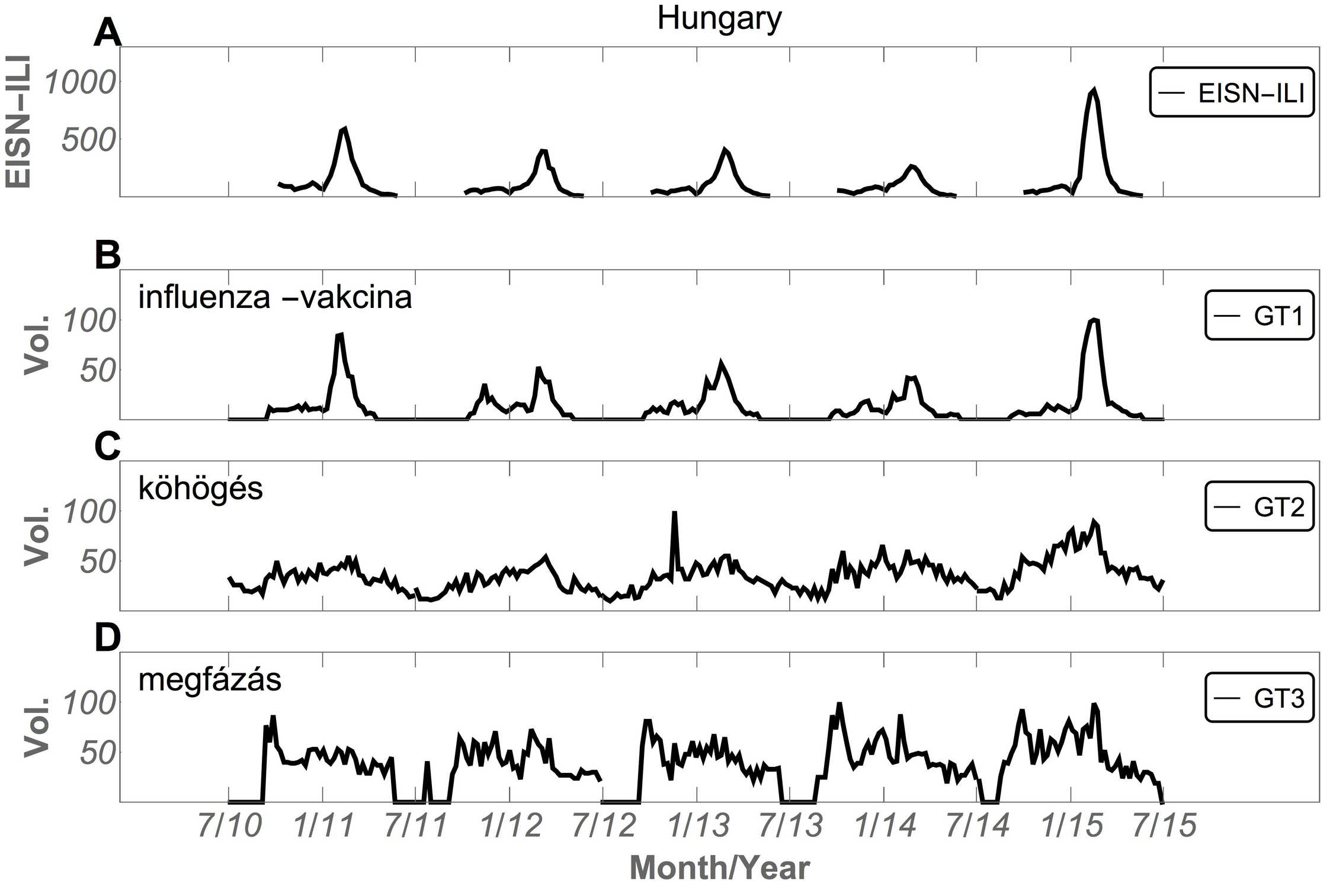

Supplement: S4 Fig — ILI rates per 100k inhabitants (A) and GT volume search (Vol.) for 3 different search terms (B to E) for Hungary for five consecutive seasons 2010–2014. The word “láz” had no significant search volume and was not included. GT time series normalized by Google, and the maximum search volume for each term is set to 100. (TIF) [file pcbi.1005330.s004.tif]

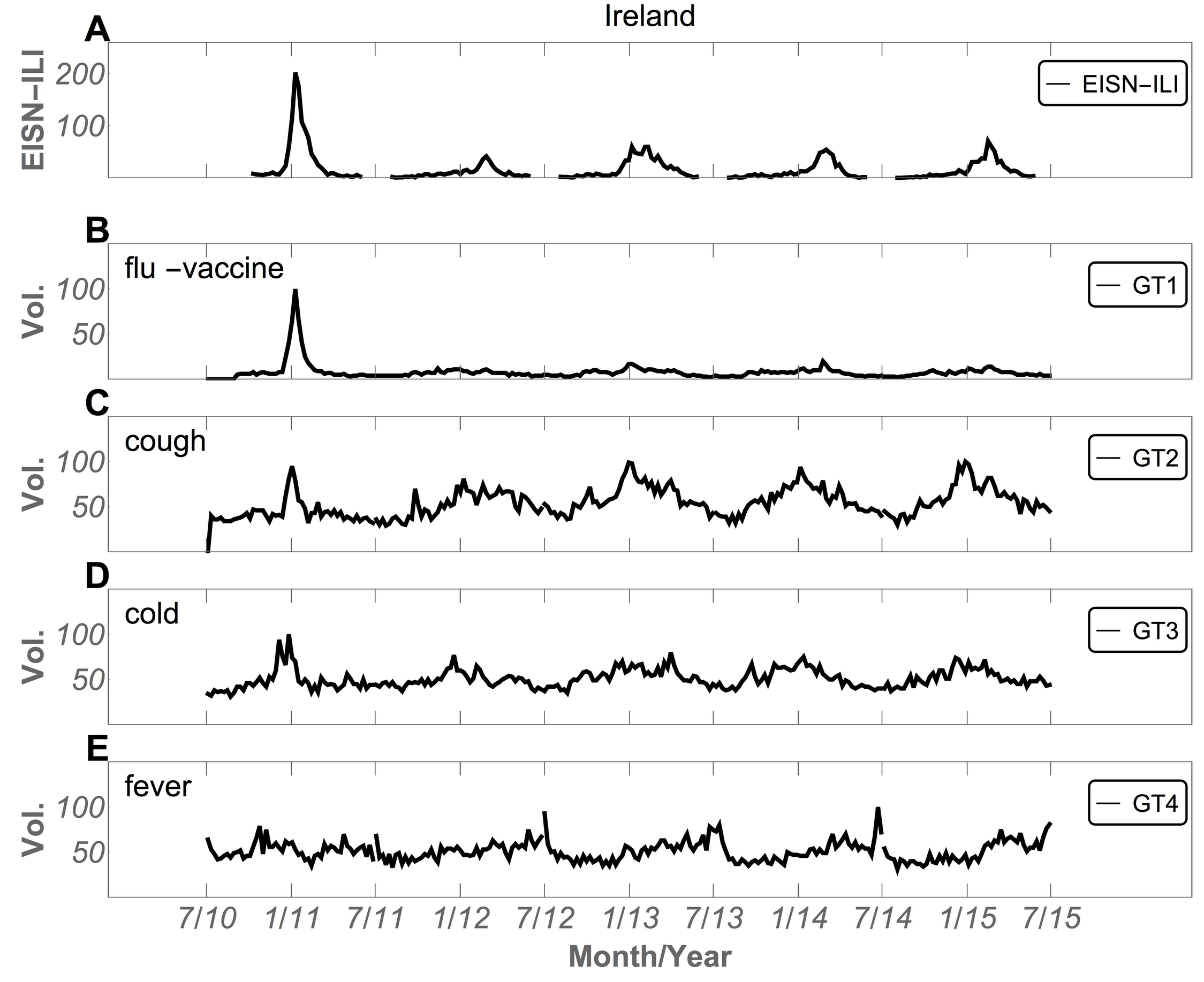

Supplement: S5 Fig — ILI rates per 100k inhabitants (A) and GT volume search (Vol.) for 4 different search terms (B to E) for Ireland for five consecutive seasons 2010–2014. GT time series normalized by Google, and the maximum search volume for each term is set to 100. (TIF) [file pcbi.1005330.s005.tif]

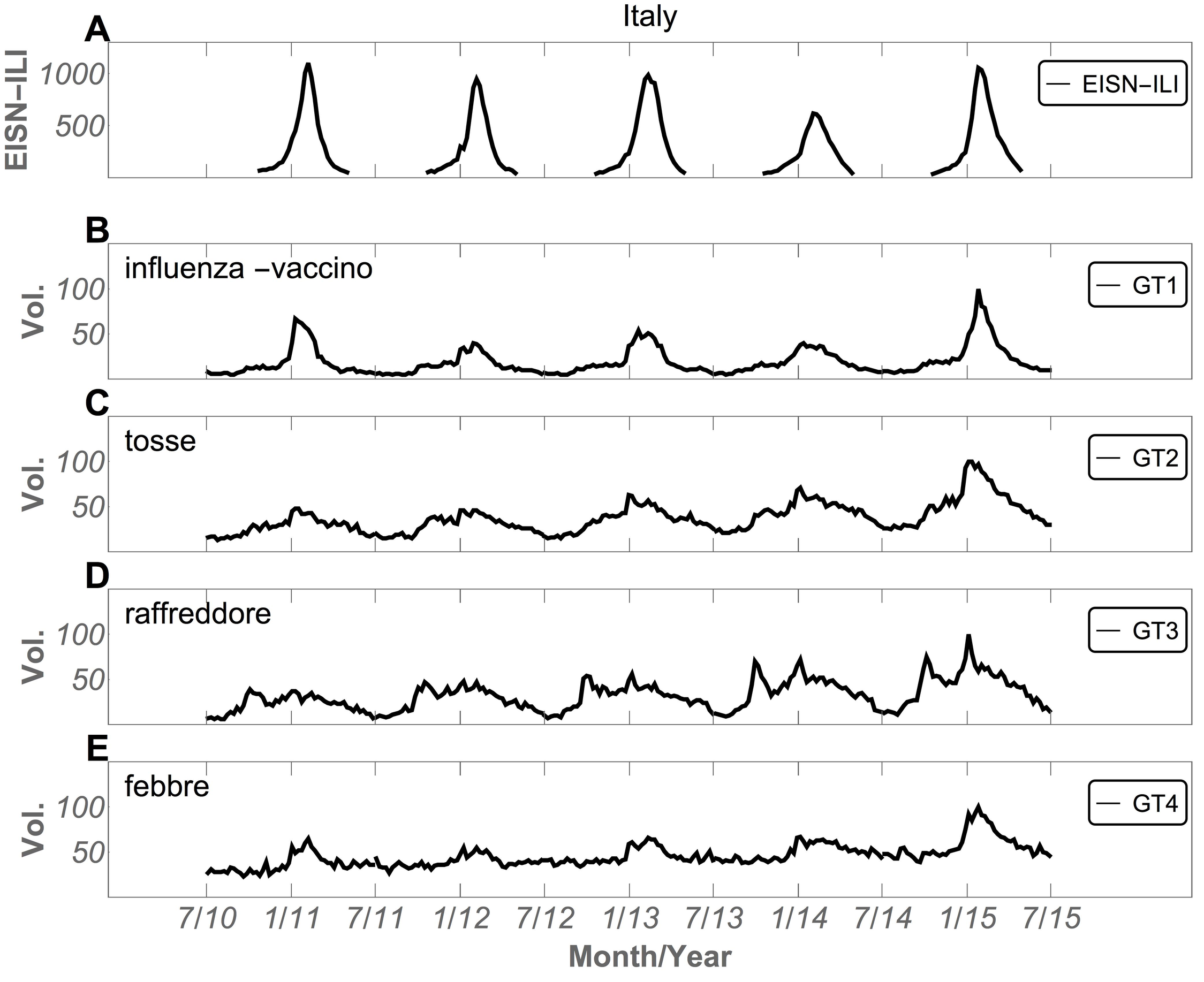

Supplement: S6 Fig — ILI rates per 100k inhabitants (A) and GT volume search (Vol.) for 4 different search terms (B to E) for Italy for five consecutive seasons 2010–2014. GT time series normalized by Google, and the maximum search volume for each term is set to 100. (TIF) [file pcbi.1005330.s006.tif]

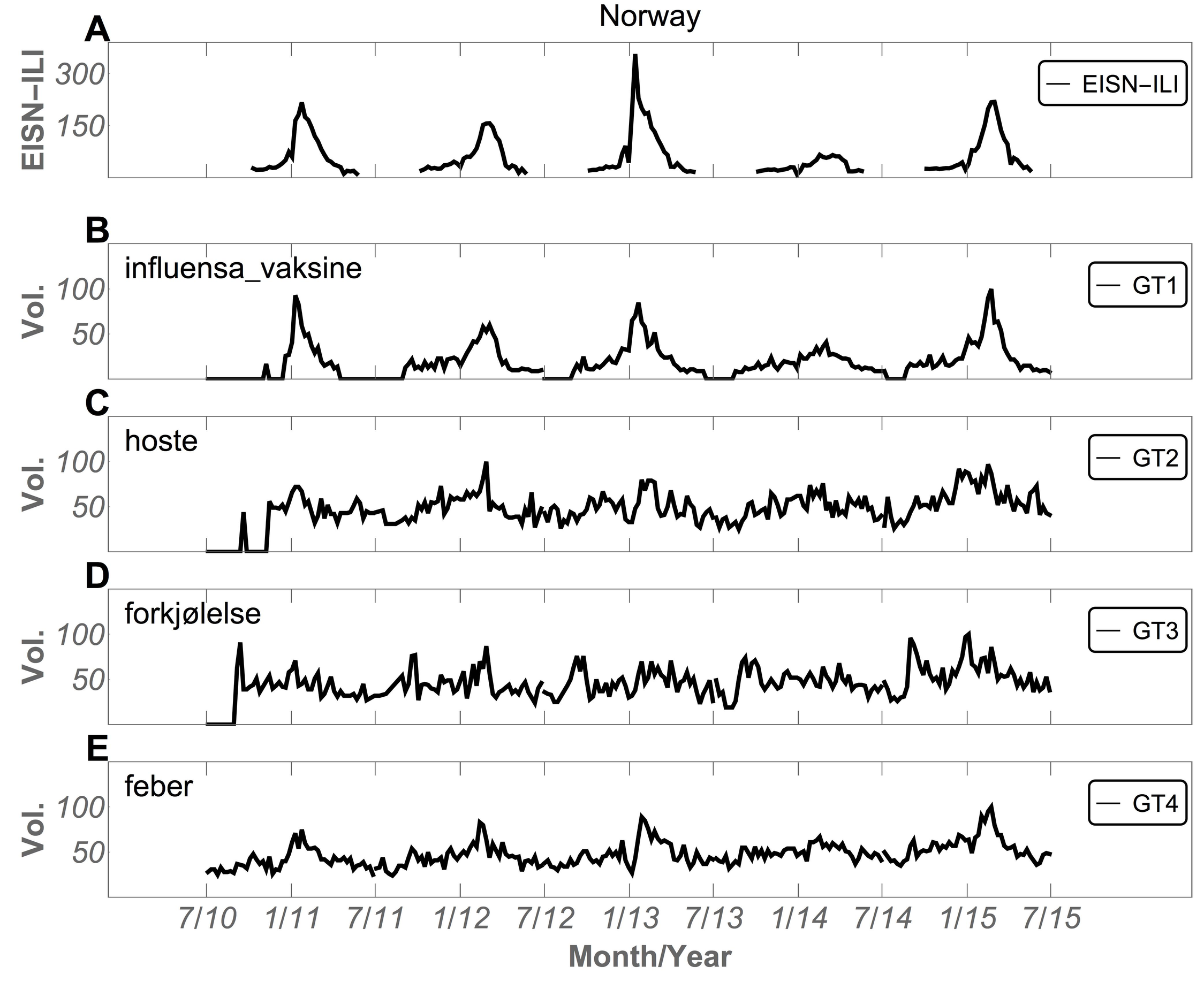

Supplement: S7 Fig — ILI rates per 100k inhabitants (A) and GT volume search (Vol.) for 4 different search terms (B to E) for Norway for five consecutive seasons 2010–2014. GT time series normalized by Google, and the maximum search volume for each term is set to 100. (TIF) [file pcbi.1005330.s007.tif]

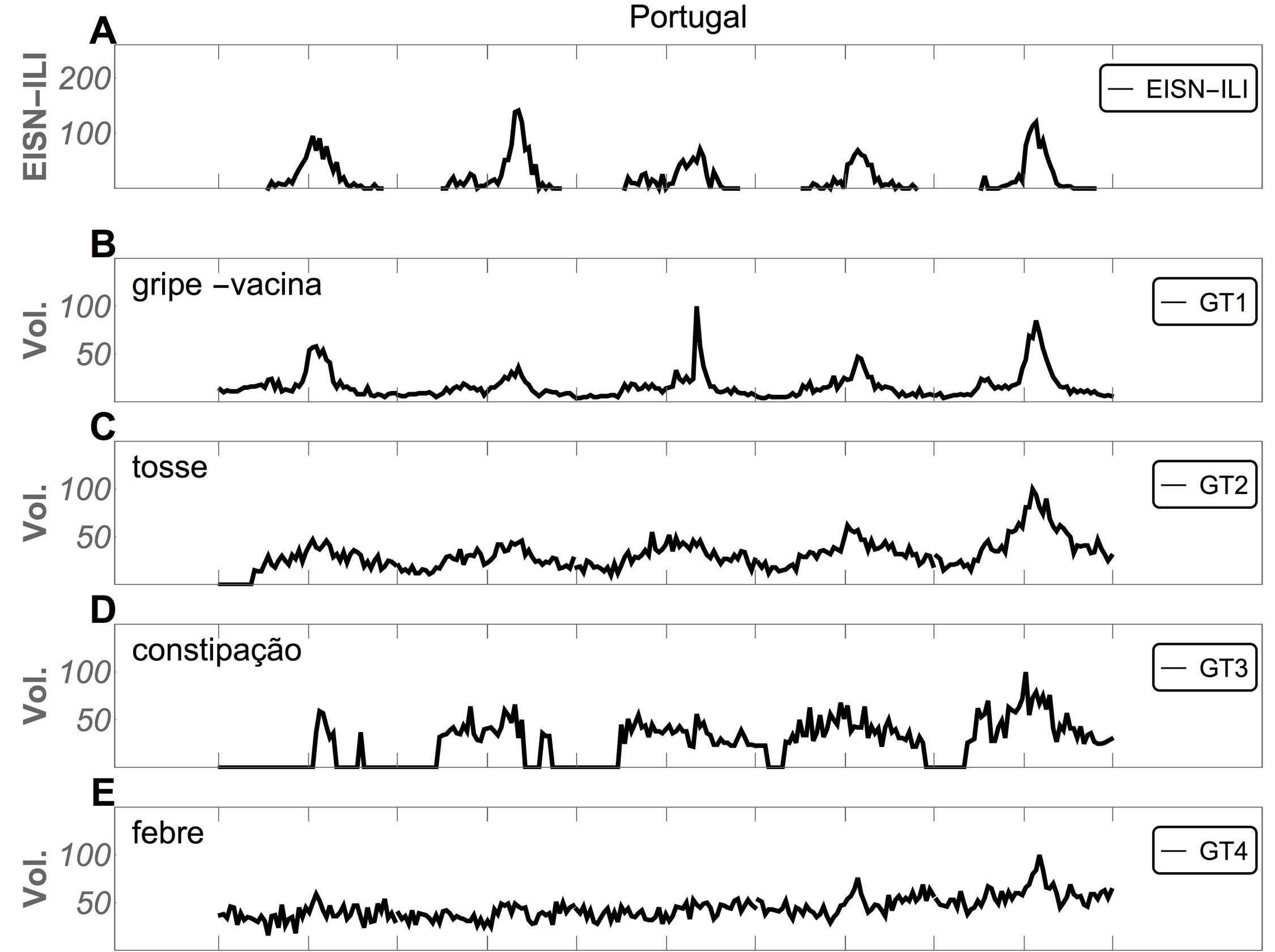

Supplement: S8 Fig — ILI rates per 100k inhabitants (A) and GT volume search (Vol.) for 4 different search terms (B to E) for Portugal for five consecutive seasons 2010–2014. GT time series normalized by Google, and the maximum search volume for each term is set to 100. (TIF) [file pcbi.1005330.s008.tif]

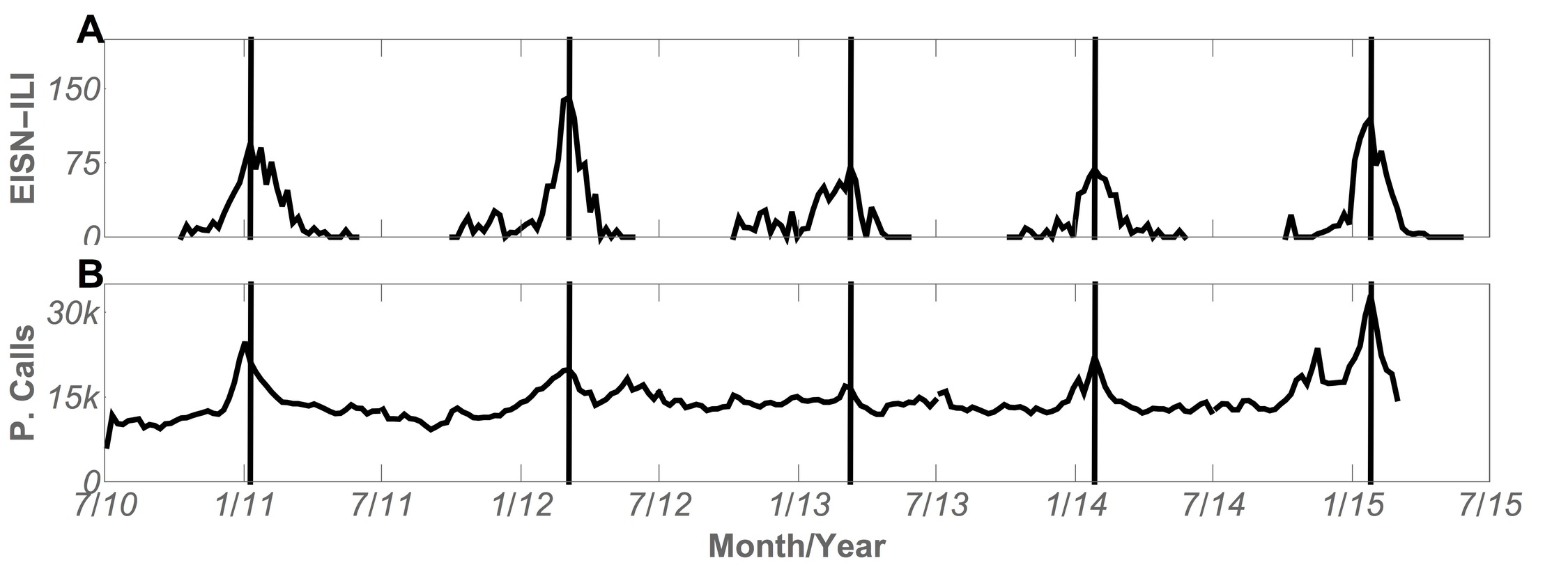

Supplement: S9 Fig — ILI rates per 100k inhabitants (A) for Portugal and absolute number of phone calls received by S24 services (B) between June/2010 and February/2015. (TIF) [file pcbi.1005330.s009.tif]

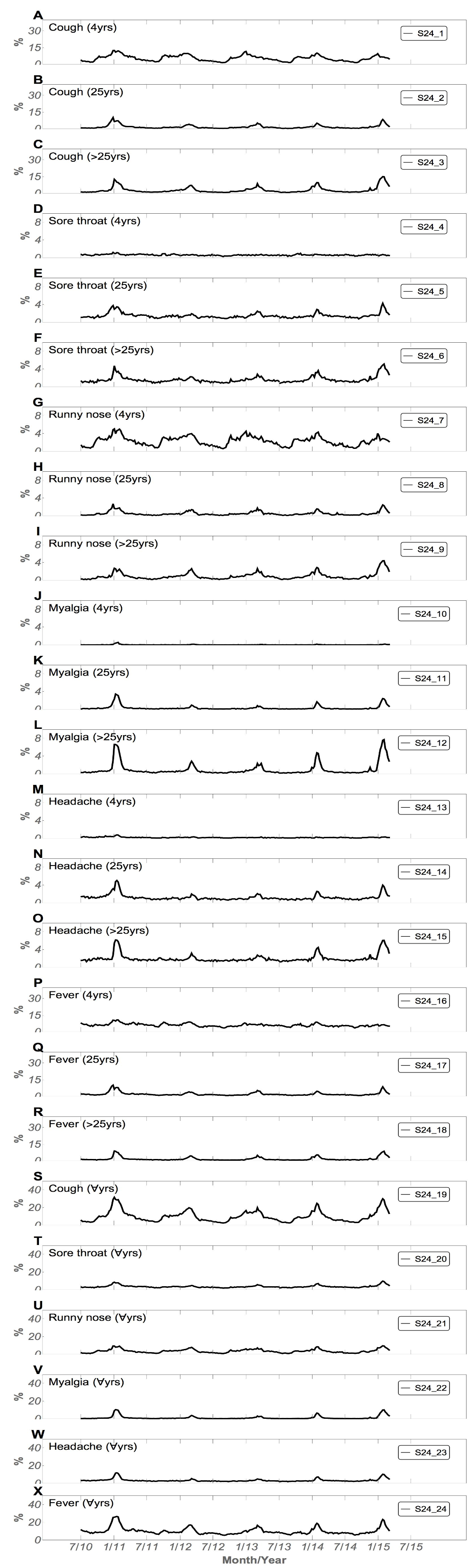

Supplement: S10 Fig — Percentage of occurrences (number of calls) of each term or related term (see S3 Table) in respect to the total number of calls received in the same considered period (one full week). (TIF) [file pcbi.1005330.s010.tif]

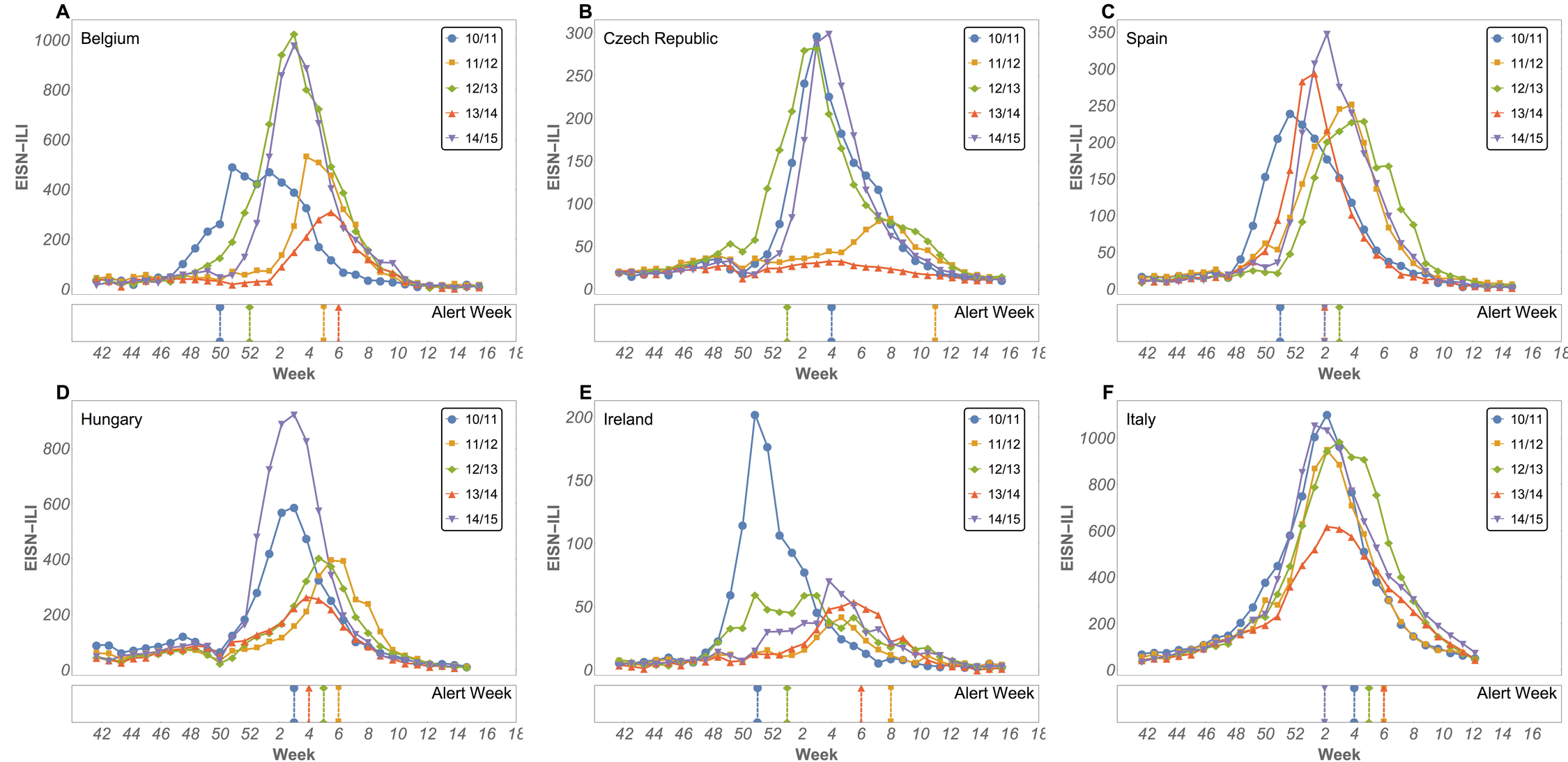

Supplement: S11 Fig — ILI rates per 100k inhabitants (top panels) for Belgium (A), Czech Republic (B), Spain (C), Hungary (D), Ireland (IE) and Italy (F), for five consecutive seasons 2010–2014. When available, the corresponding week of the official alert report is shown in the bottom panel. (TIF) [file pcbi.1005330.s011.tif]

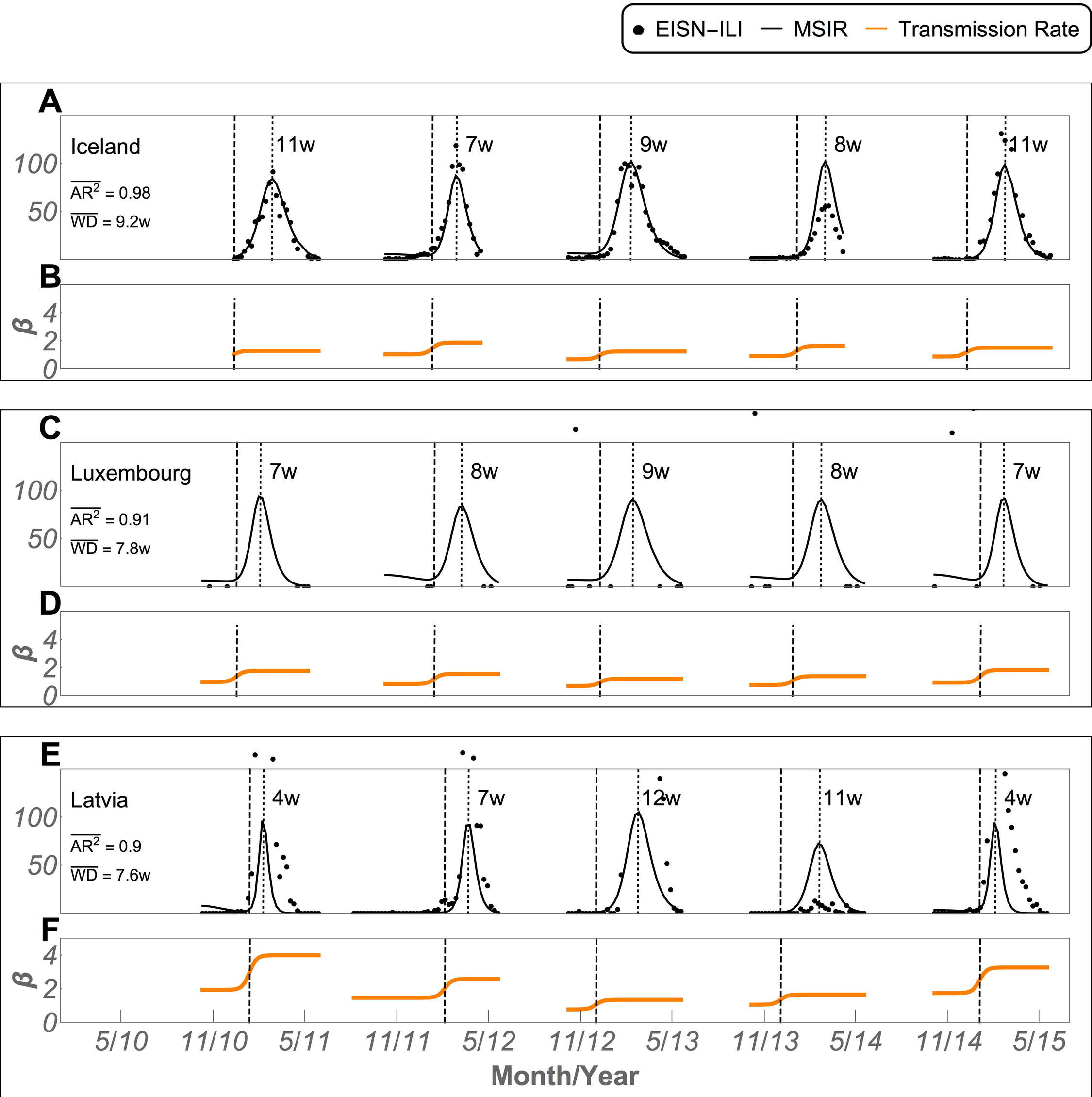

Supplement: S12 Fig — Panels A, C and E show the best MSIR fitting results and averaged AR2, where a rescaled ILI rate with the season peak set to 100 was used. Panels B, D, and F show the corresponding transmission rate (β). Dashed vertical lines connecting both panels show the transmission rate inflection point. The week difference (WD) between the infection point and the maximum MSIR fit is shown for each season and its average shown on the left of the top panels. (TIF) [file pcbi.1005330.s012.tif]

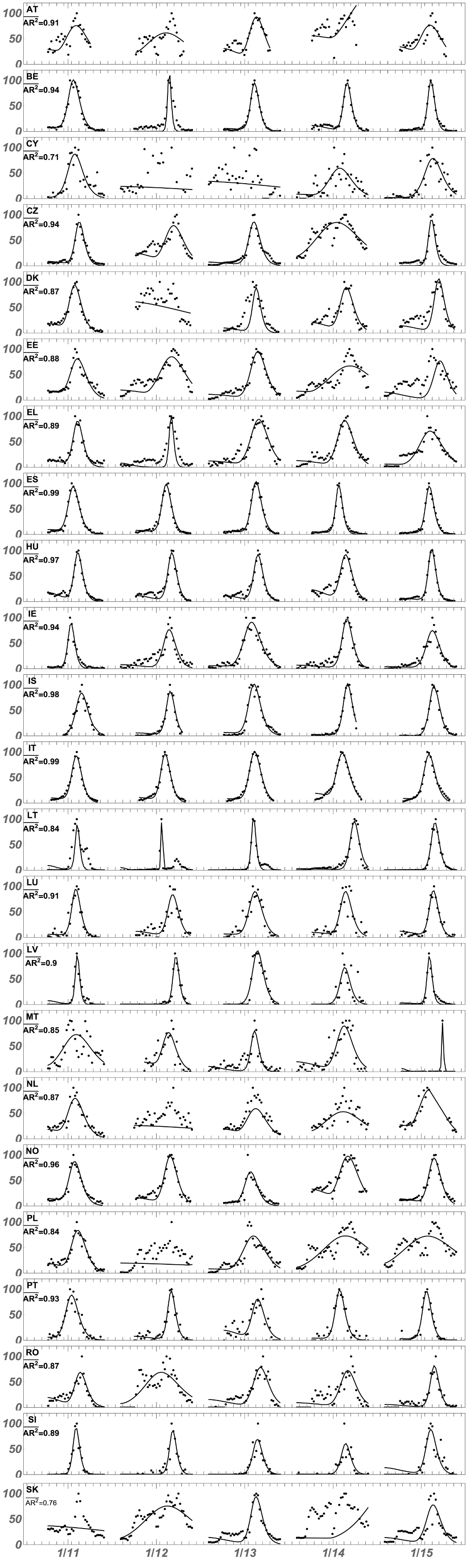

Supplement: S13 Fig — The fitting curves and averaged AR2 for all 23 countries for which we could collect EISN consistent data (see S1 Table). On the left, show the best MSIR fitting results and averaged AR2, where a rescaled ILI rate with the season peak set to 100 was used. (TIF) [file pcbi.1005330.s013.tif]

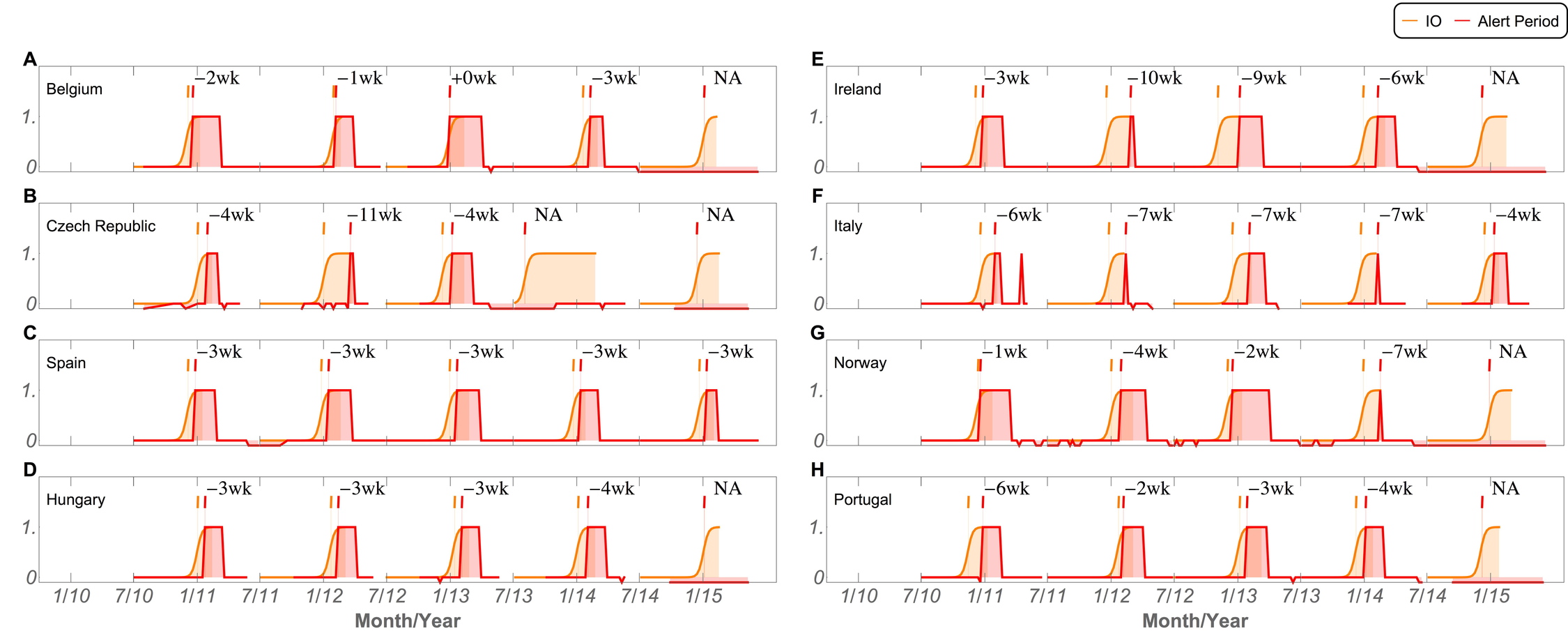

Supplement: S14 Fig — Identified Onset (IO, orange line) and official alert period (red shade) for all seasons (x-axis) and countries (panels A to H). Timing of the IO and the Alert are shown at the top, by season, in orange and red, respectively, with week differences in black. A minus sign means that the IO anticipated the Alert and a plus sign means that the IO was delayed in comparison with the official alert. NA means that at the time of collection, no official alert was available. (TIF) [file pcbi.1005330.s014.tif]

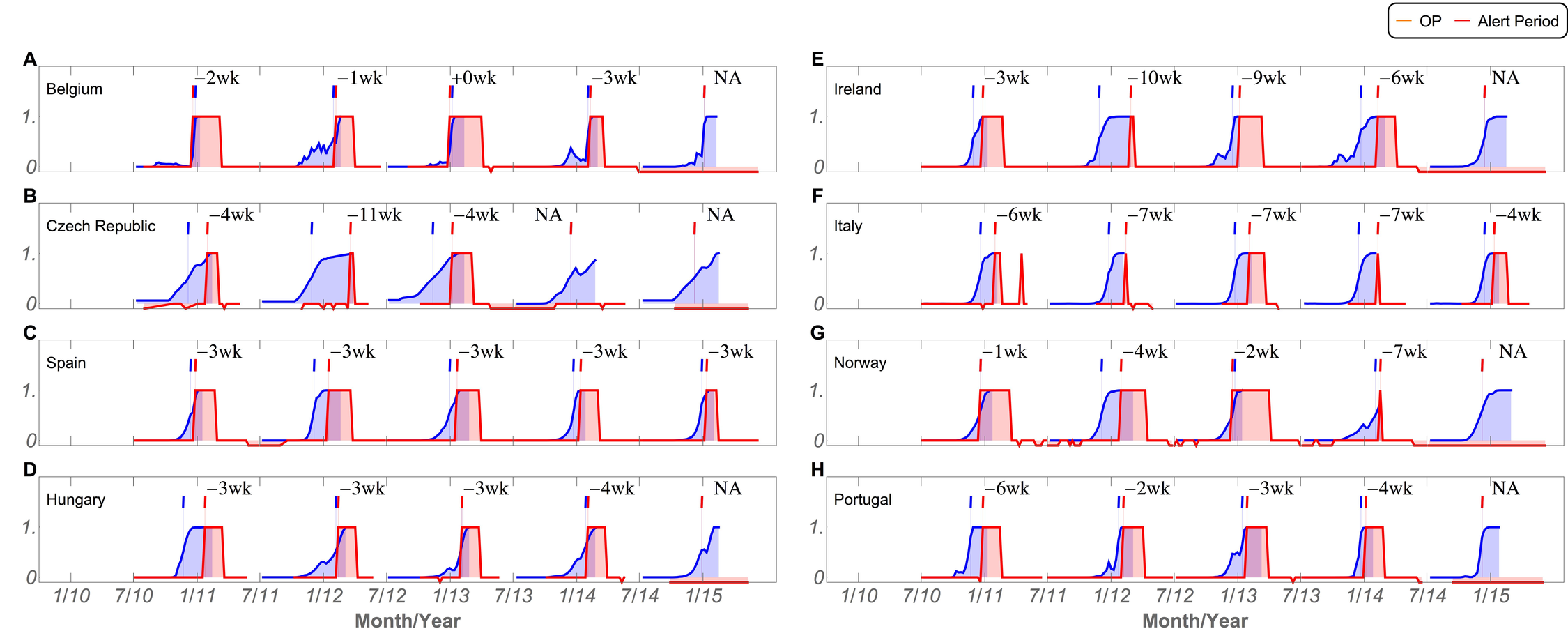

Supplement: S15 Fig — Predicted Onset (PO, blue line) and the official alert period (red shade) for all seasons (x-axis) and countries (panels A to H). Timing of the PO and the Alert are shown at the top, by season, in blue and red, respectively, with week differences in black. A minus sign means that the PO anticipated the Alert and a plus sign means that the PO was delayed in comparison with the official alert. NA means that at the time of collection, no official alert was available. (TIF) [file pcbi.1005330.s015.tif]

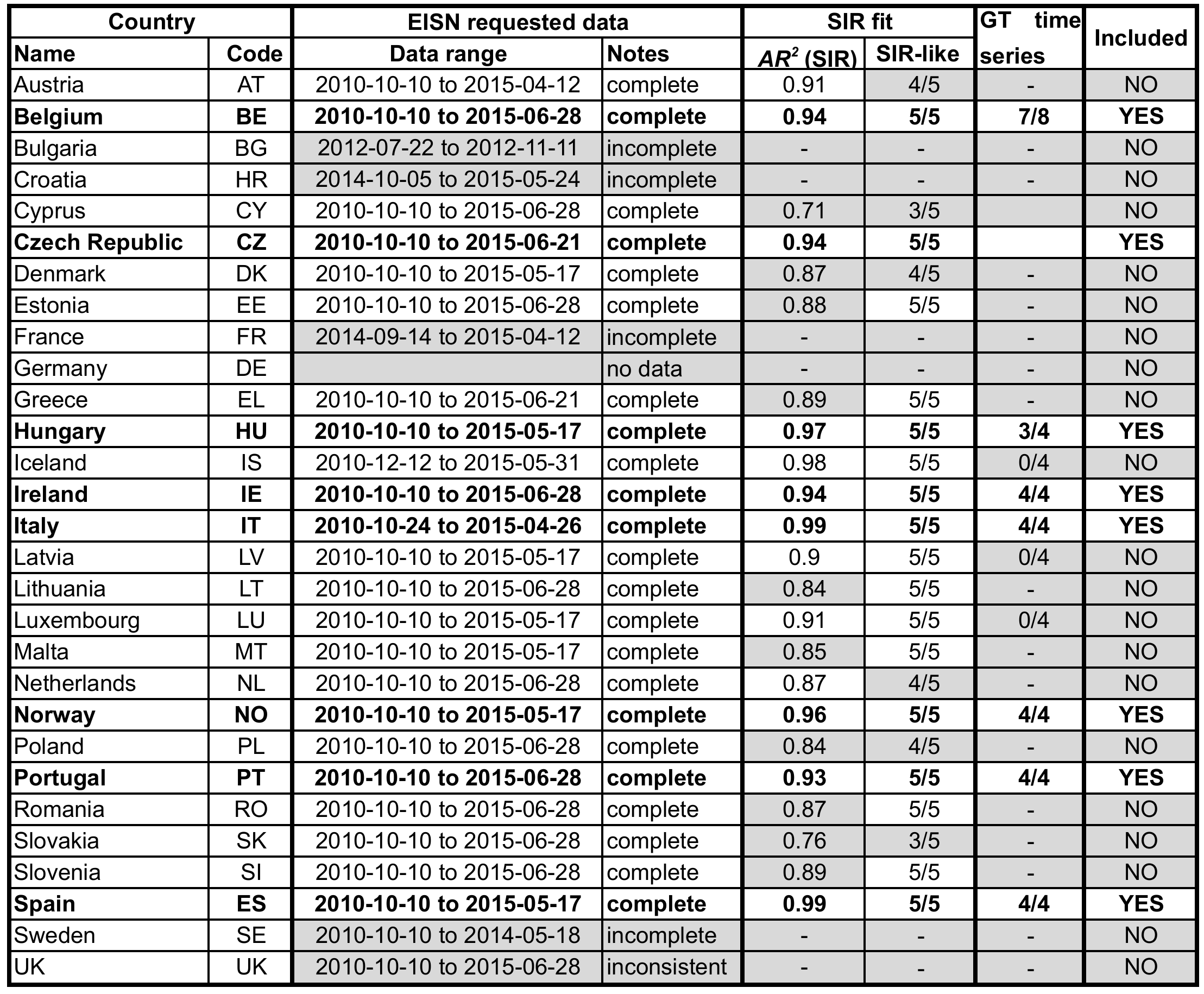

Supplement: S1 Table — Influenza data was requested from the EISN [22] on 23/06/2015, from the 2010/2011 to the 2014/2015 seasons, for all listed 29 countries. Google Trends (GT) data was retrieved on 3/09/2015 from [23]. The table shows (from column 1 to column 7): countries’ names; official country codes; data range of data received from the EISN; whether the data was “complete” (5 full seasons), “incomplete” (less than 5 seasons) or “inconsistent” (in the case of the UK the same week could have 1, 2 or 3 entries); the Averaged AR2 of the MSIR fit; how many seasons presented convergent fits; for how many Google Trends search terms we could obtain time series; whether the country data fulfilled all the inclusion criteria. Countries were not considered in the analysis if we could not collect 5 consecutive flu seasons (eliminating BG, HR, FR, DE, SE and UK), if the Averaged AR2 < 0.9 or if not all 5/5 seasons showed a convergent SIR-like shaped fit (eliminating AT, CY, DK, EE, EL, LT, MT, NL, PL, RO, SK and SI), and if at least 3/4 of the tested GT search-terms had enough search-volume to generate a time-series (eliminating IS, LV and LU). Criteria that were not full-filled are marked as grey cells. Only the countries for which the entire row is white (also bolded) were accepted. These are BE, CZ, HU, IE, IT, NO, PT and ES. (TIF) [file pcbi.1005330.s016.tif]

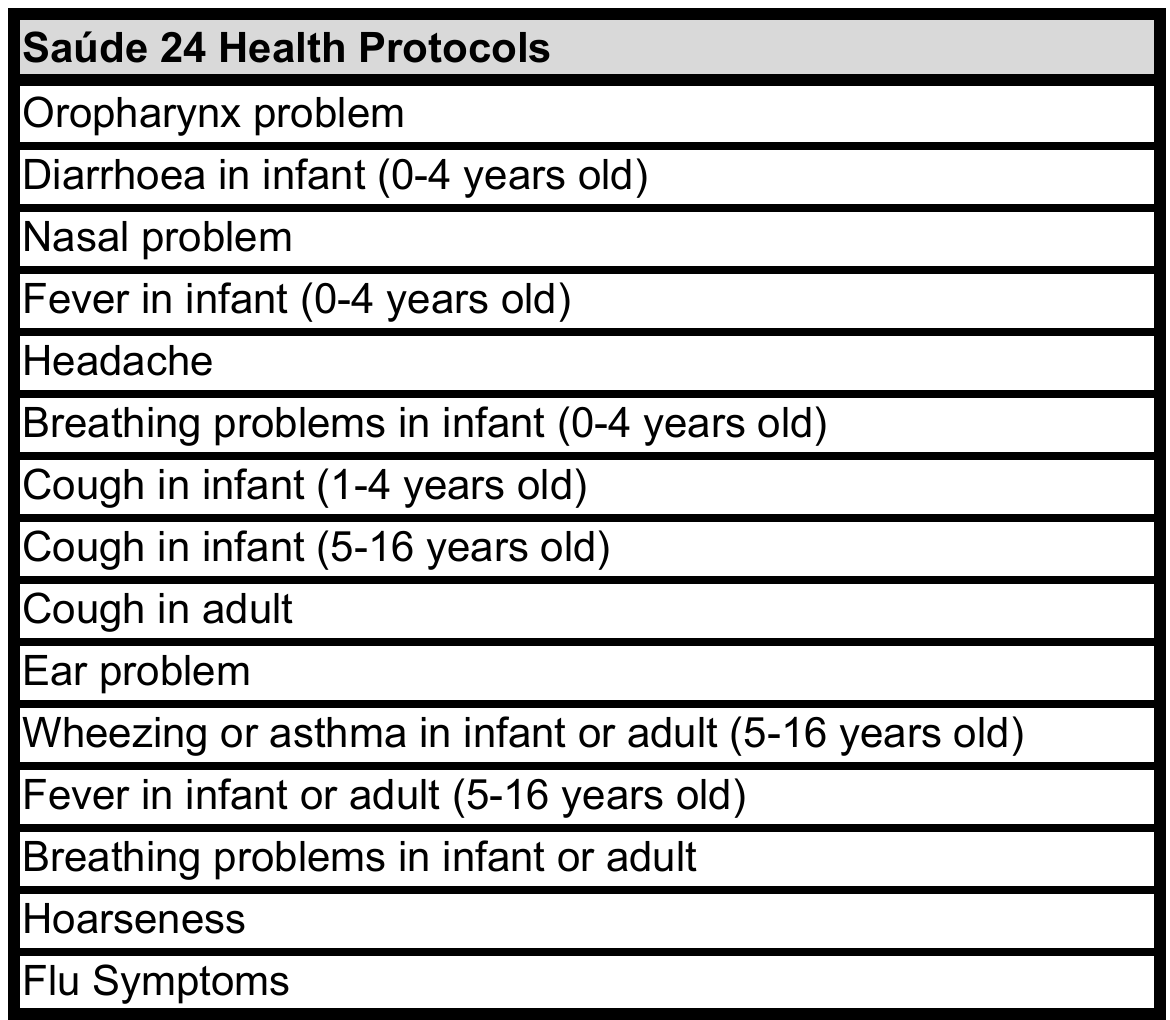

Supplement: S2 Table — Time Series of S24 calls. Occurrences of each term or related term (see S3 Table) were counted and plotted as time series, with one call corresponding to only one event. The caller’s age was divided in four age groups: 0–4 (labeled 4yrs), 5–24 (labeled 25yrs), above 25 years (labeled >25yrs) and a fourth time series with all phone logs, regardless of age (labeled)” years. The boxes on the right show the time series names, according to S3 Table. (TIF) [file pcbi.1005330.s017.tif]

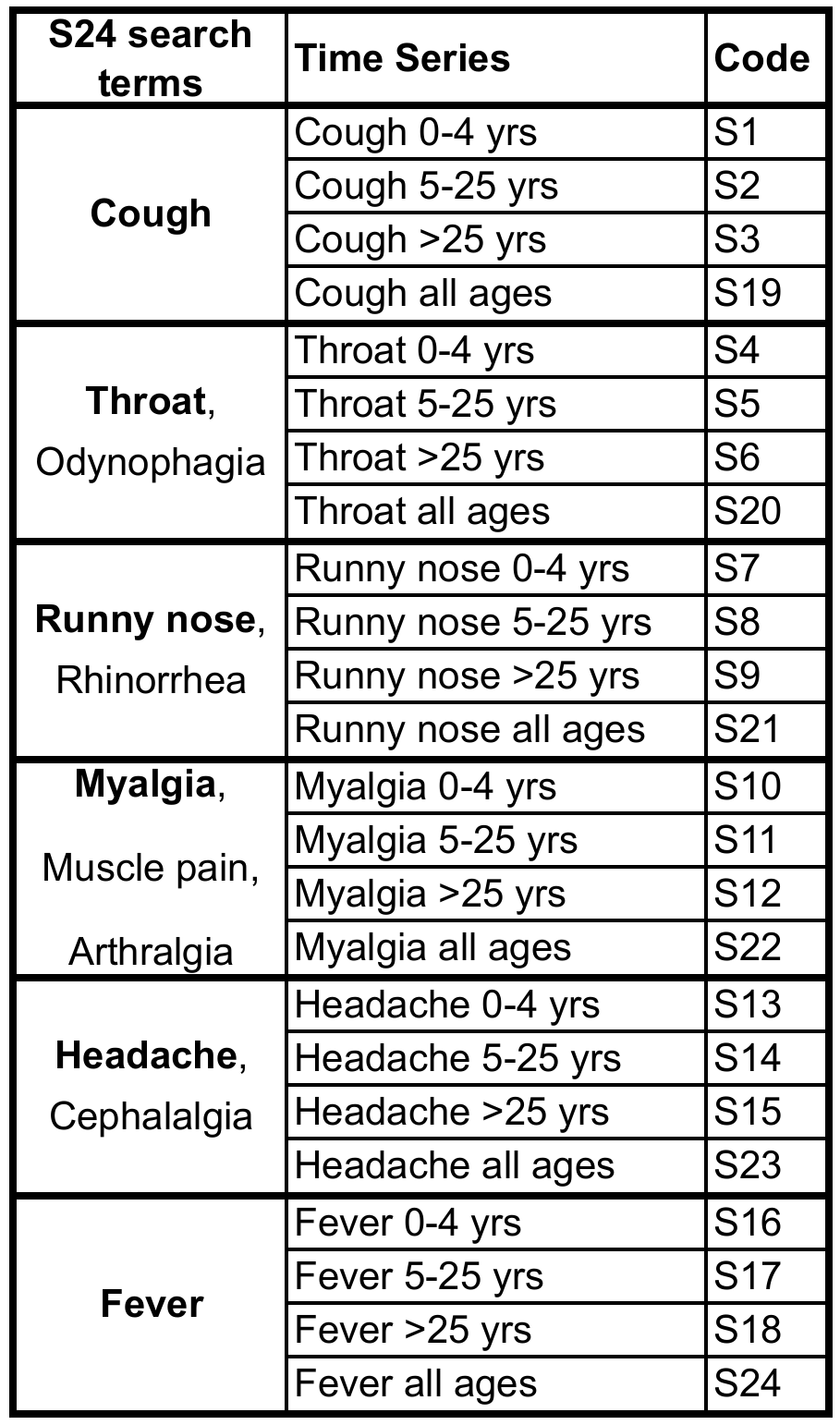

Supplement: S3 Table — From the chosen protocols (shown in S2 Table) all phone calls that included at least one of the eleven words shown in the first column were selected. These words were grouped into six “general ILI symptoms”, in bold. These were further divided into four age groups, giving rise to twenty four time series (second column), numbered as shown in the third column. (TIF) [file pcbi.1005330.s018.tif]

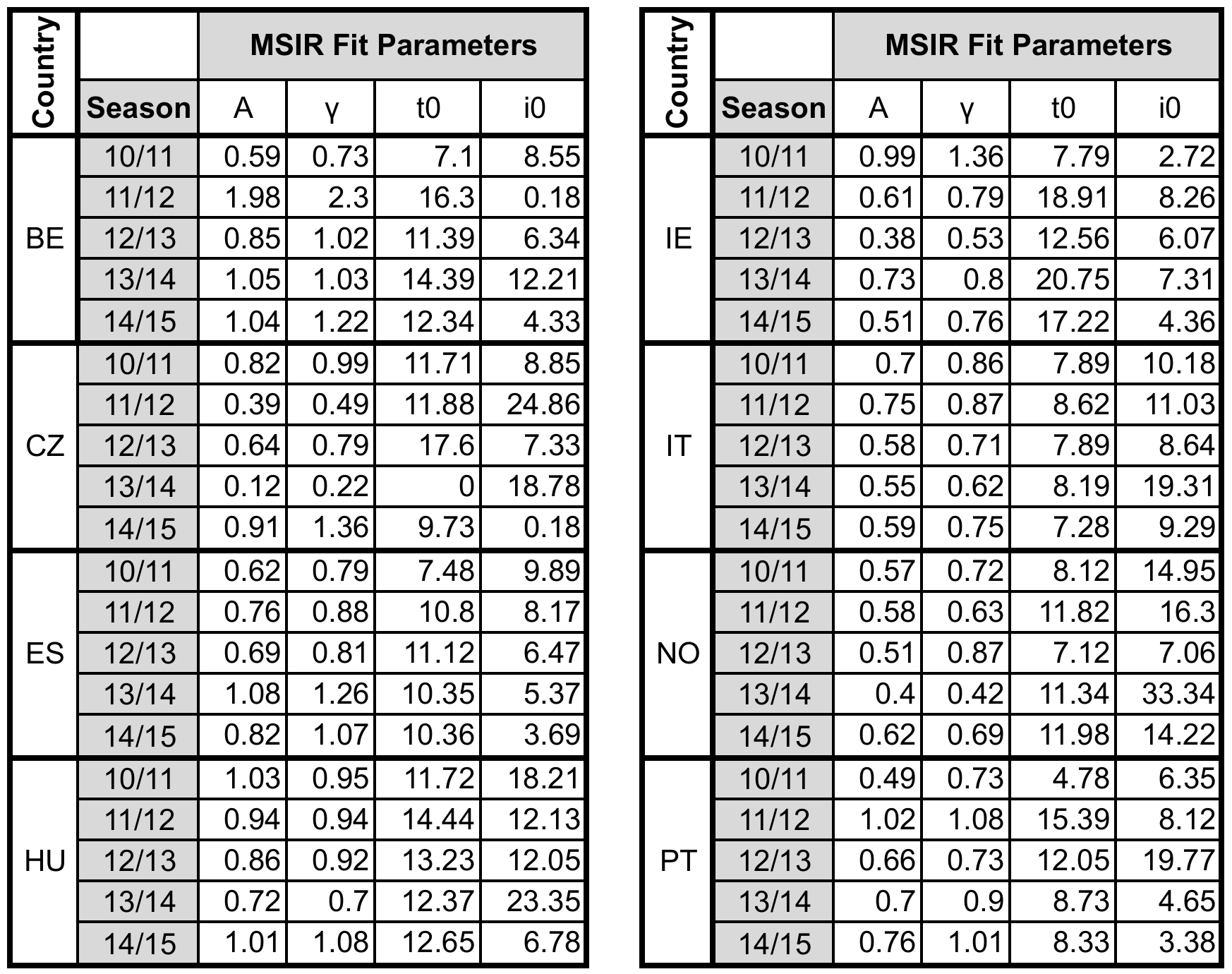

Supplement: S4 Table — Best fit parameters resulted from the MSIR non-linear fit applied to the respective ILI rates. (TIF) [file pcbi.1005330.s019.tif]

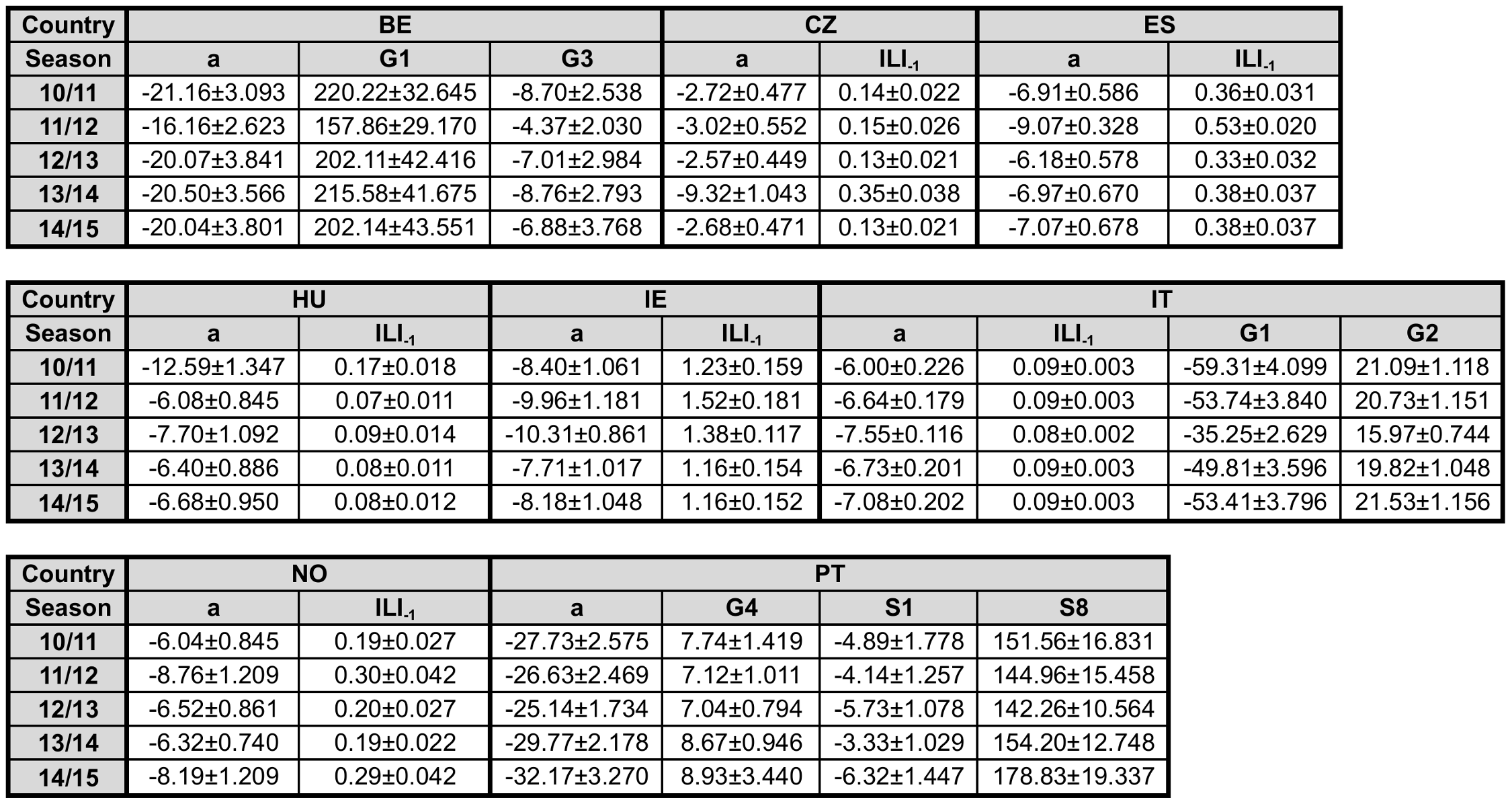

Supplement: S5 Table — Best fitted {a, bk} set for Belgium, Czech Republic, Spain, Hungary, Ireland, Italy, Norway and Portugal, for five consecutive seasons 2010–2014. (TIF) [file pcbi.1005330.s020.tif]
